# Diabetic retinopathy: shaped by the “hidden hand” of post-translational modifications

**DOI:** 10.1186/s13024-026-00973-x

**Published:** 2026-07-17

**Authors:** Ruihan Xiao, Meixia Zhang

**Affiliations:** 1 The Department of Ophthalmology, West China Hospital, Sichuan University, Chengdu, 610041 China; 2 The Department of Ophthalmology and Research Laboratory of Macular Disease, West China Hospital, Sichuan University, Chengdu, 610041 China

**Keywords:** Post-translational modifications, Diabetic retinopathy, Neurodegenerative disease, Cellular signaling, Therapeutic targets

## Abstract

Diabetic retinopathy (DR), a leading cause of vision loss in working-age adults, is increasingly recognized as a combination of neurodegenerative and microvascular disease of the retina. Its pathological mechanism is complex, involving the regulation of multiple processes such as abnormal neovascularization, nerve damage, oxidative stress, and chronic inflammation. As a key molecular mechanism for regulating protein function, post-translational modifications (PTMs) can dynamically modulate the activity and function of core cells (including retinal endothelial cells, Müller cells, and pericytes) and related signaling molecules including various forms (e.g., phosphorylation, glycosylation, acetylation, methylation, ubiquitination, and SUMOylation), thereby deeply participating in the pathological progression of DR. In addition, PTMs play a crucial role in the regulating signaling pathways, including PI3K/Akt, MAPK, AMPK, NF-κB and JAK/STAT, in the development of DR. Moreover, increasing evidence demonstrates that targeting PTMs with small molecules, either as inhibitors or activators, can reverse protein misfolding and preserve neuroretinal integrity to halt the progression of DR. This review systematically summarizes the variation characteristics of different types of PTMs in DR, analyzes the involvement of PTMs on signaling cascade and cellular processes, reviews the association between ageing and PTMs, and sorts out the research progress of PTMs as potential diagnostic biomarkers and neuroprotective therapeutic targets for DR. It aims to offer a theoretical foundation for in-depth understanding of DR pathogenesis and the development of novel prevention and treatment strategies.

## Introduction

**Fig. 1:**
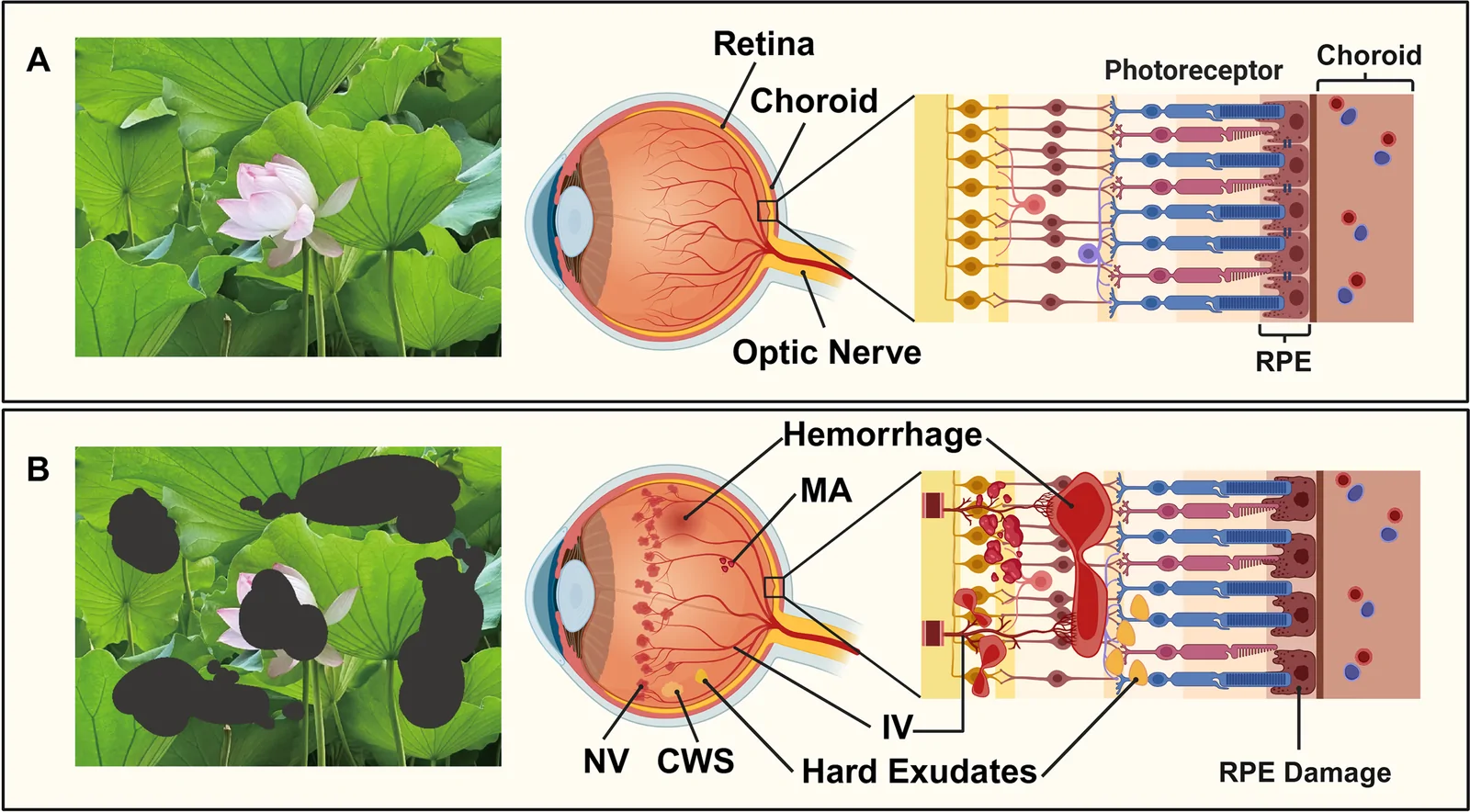
Changes of diabetic retinopathy in Vision and Retinal Anatomy. **A**) Normal vision by healthy eyes is shown in the left panel. And the schematic of healthy eye anatomy is shown in the right panel. **B**) The left panel shows DR causes visual blurring and black patches throughout the visual field. The right panel indicates general clinical symptoms of DR, including hemorrhages, microaneurysms (MA), neovascularization (NV), “cotton-wool” spots (CWS) and hard exudates. Hemorrhaging from the inner retinal vessels (IV) of the neural retina is described

As a microvascular complication of diabetes, diabetic retinopathy (DR) has been recognized for centuries. Most patients develop retinopathy within 10 to 15 years of a diabetes diagnosis [1]. By 2045, 629 million people are expected to have diabetes globally [2]. In China, the diabetes prevalence has risen from under 1% in 1980 to 11.6% in 2013 (114 million cases) [2]. As diabetes prevalence rises, more individuals are at risk for retinopathy. It has been reported that DR has caused 3.7 million cases of visual impairment and over 833,000 cases of blindness [3], making it the primary cause of preventable blindness in working-aged people [4, 5]. The physiological layered organization of the retina ensures normal phototransduction and visual processing, while DR disrupts this structure through vascular and degenerative changes such as microaneurysms (MA), intraretinal vascular abnormalities, cotton wool spots (CWS), neovascularization (NV), retinal hemorrhage, and hard exudates, accompanied by RPE and photoreceptor damage that leads to visual loss (Fig. 1). Given the disease burden, it is of utmost importance to explore the DR pathogenesis to provide novel approaches for efficient and accurate diagnosis, risk assessment, and treatment.

To date, a wide range of pathological events have been found to drive DR progression, including an imbalance in neuroprotective factors production and extracellular glutamate buildup [6], protein kinase C (PKC) activation [7], oxidative stress [8], accumulation of advanced glycation end products (AGEs) [9], inflammation [10], polyol pathway activation [11], endoplasmic reticulum (ER) stress, and mitochondrial dysfunction [12]. These processes are interrelated and may all involve post-translational modifications (PTMs). PTMs are therefore considered to be a potential molecular mechanism underlying the occurrence and progression of DR.

As fundamental regulators of protein function, PTMs are widely present in cellular activities. PTMs refer to reversible or irreversible covalent processing of some proteins after translation, which occurs in amino acid side chains, C-termini, or N-termini [13]. In the different biological macromolecules, proteins exhibit the most functional and structural variations. From metabolism and catalysis to signaling and locomotion, most cellular and physiological processes are enacted by proteins, making them indispensable participants in homeostasis as well as in the development of diseases. Accumulating evidence have identified that dysregulated PTMs contribute to the pathogenesis of multiple diseases, such as cancer, neurodegenerative diseases, and diabetes [14–16]. Among these, the diabetic environment can significantly accelerate and alter protein PTMs [17–19]. Nevertheless, the connections between PTMs with relevant signaling pathways and DR pathogenesis remain poorly elucidated.

**Fig. 2:**
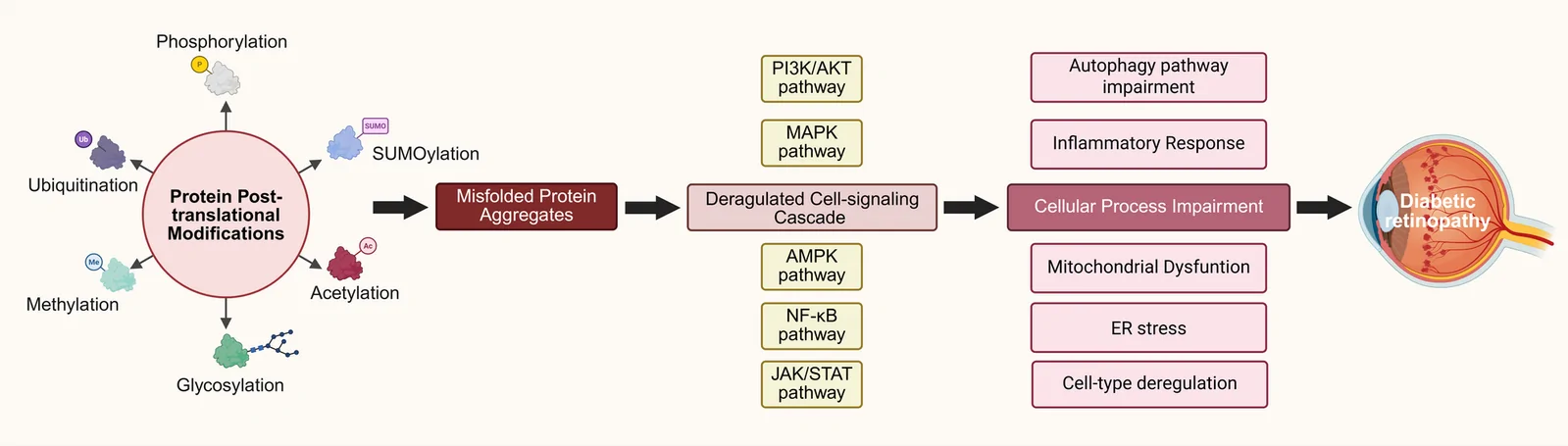
Overview on how PTM remodeling contributes to DR progression. PTMs in DR primarily include phosphorylation, O-GlcNAcylation methylation, acetylation, ubiquitination and SUMOylation. PTMs cause aggregation of misfolded proteins, which will deregulate cell-signaling cascades such as PI3K/Akt pathway, MAPK pathway, AMPK pathway, NF-κB pathway and JAK/STAT signaling cascade. Further, deregulation in the cell-signaling cascade causes impairment in cellular processes such as autophagy pathway impairment, mitochondrial dysfunction, inflammatory response, endoplasmic reticulum stress and cell-cycle deregulation, which enhances progression of DR

To better clarify the molecular mechanisms underlying DR pathogenesis, it is warranted to integrate these expanding PTM insights into a comprehensive framework. Herein, we established a comprehensive PTM-driven architecture in DR, mapping PTM alterations to specific signaling networks, cell lineages, and progressive organelle dysfunction. Specifically, we reviewed how aberrant PTMs alter the activity of vital signaling cascades within specific retinal cells, their impact on biological pathways, and potential treatment for DR resulting from aberrant PTMs (Fig. 2).

## Common types of PTM in DR

**Fig. 3:**
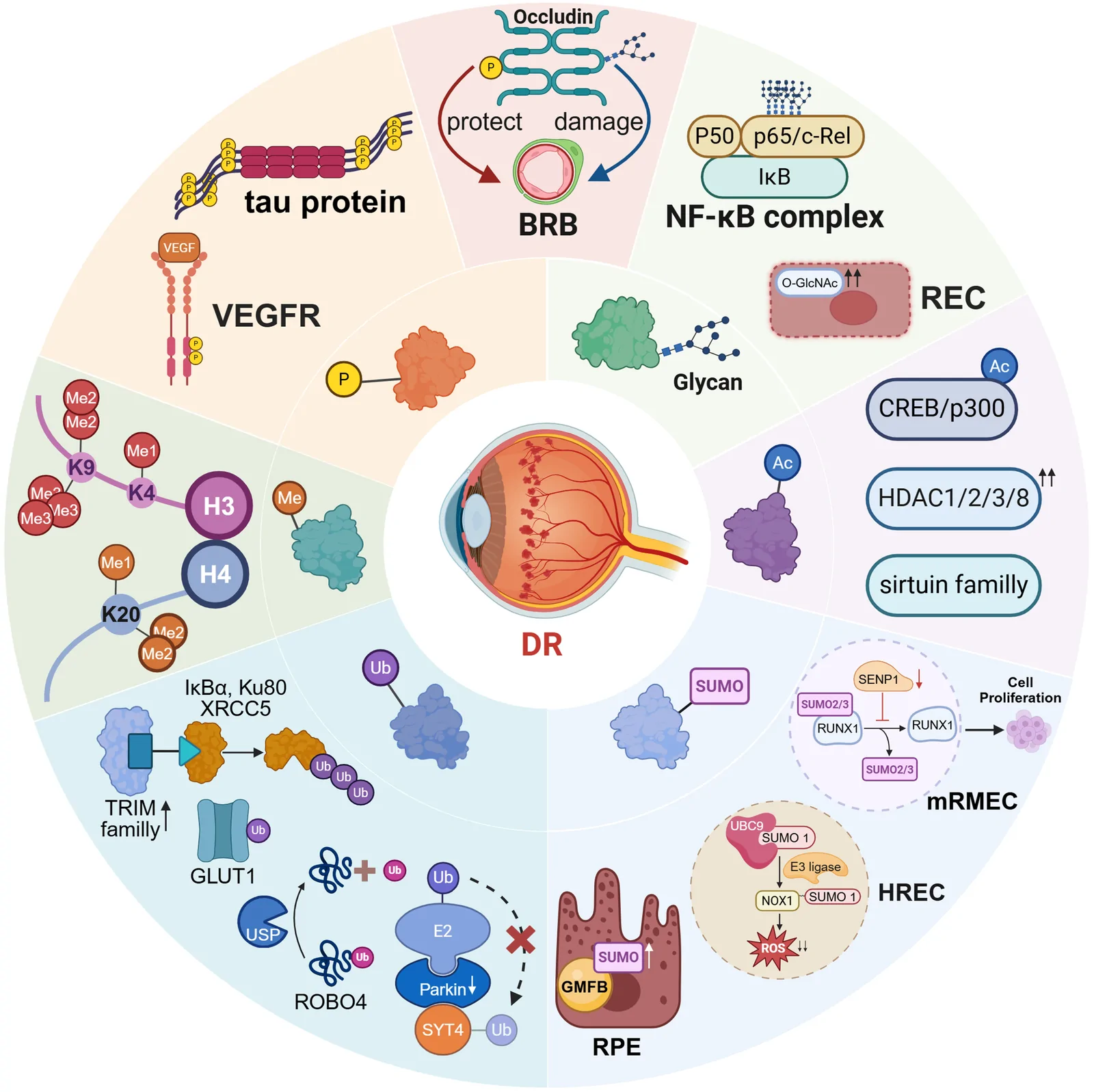
Common PTMs associated with the progression of DR. PTMs of histone and non-histone substrates were involved in the pathogenesis and progression of DR. Phosphorylation, as one of the most common PTM, is a reversible process regulated by protein kinases and phosphatases. Hyperphosphorylated tau has been detected in the RGC layer and associated with neuroretinal dysfunction, while phosphorylation of VEGFR could induce recruitment and activation of several key adaptor proteins. Protein glycosylation refers to adding glycan moieties to proteins. O*-*GlcNAc is elevated in retina endothelial cell, and increased O-GlcNAcylation of NF-κB p65 subunit is associated with RGC loss. O-GlcNAcylation and phosphorylation have been shown to participate in extensive crosstalk with each other, for example, suppressing glycosylated occludin expression can enhance its phosphorylation, thereby increasing the barrier function of tight junctions. Acetylation is the process by which the acetyl group from acetyl coenzyme A is transferred to lysine or other amino acid residues on target proteins. Hyperglycemia promotes acetylation of CBP/p300, and different isoforms of HDAC can also participate in the pathophysiology of diabetic vascular complications in retina, and sirtuin family is also involved in the development of DR. Protein methylation refers to adding methyl groups (CH₃) to the N-terminus of proteins. Monomethylation of H3K4me1 and dimethylation and trimethylation of H3K9me2/me3, and mono- and di-methylation of H4K20me1/me2 are found in DR. Protein ubiquitination means that attaches ubiquitin to substrate proteins. TRIM family exerts vital effects on a variety of biological activities in DR onsetting. USP5 could repress cell proliferation and aggravate high glucose-induced HRPE cell damage by deubiquitinating ROBO4 protein. Downregulated Parkin expression inhibits the ubiquitination and degradation of SYT4, resulting in exacerbating the progression of DR. In addition, mono- or diubiquitinated GLUT1 could protect retinal endothelia cells by decreasing glucose transport. SUMOylation indicates that catalyzing the covalent binding of SUMO proteins to lysine residues in target proteins. Enhanced SUMOylation of GMFB can be detected in RPE cells, while SENP1 are reduced, inhibiting SUMO2/3 modification, which play a negative role in DR; however, SUMO1 may suppress Nox1-controlled ROS production in DR via SUMOylation-dependent UBC9-mediated intracellular transport, which play a positive role in DR. BRB, blood-retinal barrier; VEGFR, vascular endothelial growth factor receptor; GLUT1, glucose transporter 1;ROBO4, Roundabout 4; RPE, retinal pigment epithelial; GMFB, glial maturation factor beta; ROS, reactive oxygen species; HREC, human retinal endothelia cells; MRMEC, mouse retinal microvascular endothelial cells

PTM can be divided into several categories, including the addition of functional/chemical groups (phosphorylation, acetylation, methylation, amidation, formylation), polypeptide chains (ubiquitination, SUMOylation, neddylation), complex molecules (glycation, oxidation, palmitoylation, carbamylation, PEGylation), and amino acid modification (citrullination, isoaspartate formation, racemization, proteolytic cleavage) [20]. In this part, we focused on the most common proteins modification linked to the progression of DR (Fig. 3; Table 1).

**Table 1:** Summary of major protein post-translational modifications involved in DR

Type of PTM	Target protein	Affected cells types	Pathological effects of Dysregulated PTMs in DR	Refs
Phosphorylation	Tau, VEGFR2, occludin, ZO-1, connexin43, eNOS, MeCP2, α-crystallin, myosin	RGC, retinal endothelial cells, Müller cells, pericytes	- Tau hyperphosphorylation disrupts microtubule transport and contributes to early retinal neurodegeneration; - VEGF-induced VEGFR2 autophosphorylation drives pathological angiogenesis and vascular leakage; Altered phosphorylation of occluding, ZO-1, and connexin43 impairs BRB function; - Upregulated phosphorylation of eNOS contributes to endothelial dysfunction and vascular leakage; - Phosphorylation of MeCP2 increases VEGF production, leading to neovascularization; - Decreased phosphorylation of α-crystallin promotes death of Müller glia and RGC; - Phosphorylation of myosin leads to membrane contraction, retinal tears, and detachment.	[21–42]
O-GlcNAcylation	YAP/TAZ, NF-κB p65, occludin, connexin43, Sp1, ZFRBP, Tumor Protein p53	Retinal endothelial cells, RGCs, Müller cells, pericytes	- Elevated O-GlcNAcylation activates the Hippo-YAP/TAZ pathway and promotes endothelial activation; - O-GlcNAcylation of NF-κB p65 accelerates RGC loss under hyperglycemic conditions; - GlcNAcylation crosstalk with phosphorylation to jointly regulate BRB integrity; - GlcNAcylation of Sp1 drives retinal inflammation and early vascular damage; - Elevated O-GlcNAcylation of ZFRBP and Tumor Protein p53 induces endothelial migration and tube formation, leading to neovascularization.	[35–37, 43–57]
Acetylation	CBP/p300, NF-κB p65, TXNIP, histones, Mfn2, LXRα	Retinal endothelial cells, RGC, retinal capillary cell	- Hyperglycemia enhances CBP/p300 acetylation and triggers NF-κB-mediated retinal inflammation; - Dysregulated HDAC and sirtuin family members exert opposite effects on DR progression; - Elevated acetylation of Mfn2 leads to mitochondrial dysfunction and endothelial metabolism impairment; - Increased acetylation of LXRα aggravates vascular endothelial inflammation.	[58–84]
Methylation	Histone H3R17, H3K4, H3K9, H4K20	RPE cells, retinal endothelial cells, Müller cells	- Upregulated PRMT4 induces H3R17 methylation and promotes RPE cell death; - Aberrant histone methylation elevates ROS production and leads to endothelial dysfunction; - Mono- and di-methylation states of histone H4 are upregulated in the Müller cells.	[85–95]
Ubiquitination	SYT4, IκBα, DAB1, SLC1A5, ROBO4, GLUT1, Cx43	Retinal endothelial cells, RPE cells, photoreceptors, Müller cells, RGC	- Impaired ubiquitination and degradation of SYT4 aggravates glucose uptake, apoptosis, and inflammation of RPE; - Reduced IκBα ubiquitination promotes activation of inflammatory responses in retinal endothelial cells; - Polyubiquitination of DAB1 alleviates inflammatory responses in RPE; - Deubiquitinated SLC1A5 promotes cell proliferation, and suppresses cell apoptosis, inflammation, ferroptosis, and oxidative stress; - Deubiquitinated ROBO4 high glucose-induced RPE damage; - Reduced ubiquitination of IκBα promotes activation of inflammatory responses in Müller cells; - Mono- or di-ubiquitinated GLUT1 reduced glucose transport into retinal endothelial cells; - Hyperglycemia activates the ubiquitin-proteasome system and degrades tight junction proteins; - Ang II triggers an upregulation of ubiquitin-conjugated synaptophysin expression, affecting the survival of RGC and inner retinal cells.	[96–114]
SUMOylation	GMFB, RUNX1, Nox1, Drp1, DBC1	RPE cells, retinal microvascular endothelial cells, pericytes	- Abnormal SUMOylation of GMFB promotes inflammatory responses in RPE cells; - Altered expression of SENP1/3 disrupts SUMO balance and exacerbates retinal damage; - SUMO1 plays a protective role via suppressing Nox1-mediated oxidative stress; - DeSUMOylation of Drp1 disrupts mitochondrial dynamics and apoptosis in retinal endothelial cells; SUMOylated DBC1 contributes to pericyte loss.	[115–122]

DR, diabetic retinopathy; PTM, post-translational modifications; VEGFR2, vascular endothelial growth factor 2; ZO-1, zonula occluden-1; eNOS, endothelial nitric oxide synthase; MeCP2, methyl-CpG-binding protein 2; RGC, retinal ganglion cells; YAP, Yes-associated protein; TAZ, Transcriptional co-activator with PDZ-binding motif; NF-κB, Nuclear Factor kappa-light-chain-enhancer of activated B cells; ZFRBP, zinc finger RNA-binding protein; BRB, blood-retinal barrier; TXNIP, thioredoxin-interacting protein; Mfn2, Mitofusin 2; LXRα, liver X receptor α; RPE, retinal pigment epithelium; HDAC, histone deacetylase; H3R17, histone H3 at arginine 17; ROS, reactive oxygen species; SYT4, synaptophysin 4; IκBα, inhibitor of nuclear factor kappa B alpha; ROBO4, Roundabout 4; GLUT1, glucose transporter 1; Ang II, angiotensin II; UPS, ubiquitin–proteasome system; GMFB, glial maturation factor beta; RUNX1, RUNX family transcription factor 1; DBC1, deleted in breast cancer 1; SENP, SUMO specific peptidase

### Phosphorylation

Phosphorylation, one of the most common PTM, is a reversible process regulated by protein kinases and phosphatases. Specifically, it is the process in which phosphate groups bind to substrates under the regulation of protein kinases, thereby modulating protein activity and interactions [21]. It is deeply involved in regulatory processes, including membrane transport, enzyme activity, and protein degradation and interactions [22]. Therefore, phosphorylation plays a crucial role in regulating apoptosis, oxidative stress, inflammation, cellular signaling, translocation, mitochondrial function, and autophagy [23–26].

Within the complex multicellular environment of the diabetic retina, abnormal phosphorylation events do not act uniformly, but rather exhibit distinct, cell-type-specific pathological features across microvascular network and neural retina. Growing evidence suggests that synaptic neurodegeneration of retinal ganglion cells (RGCs) and activation of glial cells may be among the earliest events in DR pathogenesis [27, 28]. Recently, hyperphosphorylated tau (the microtubule-associated protein) has been found in the RGC layer of transgenic mice carrying the P301S mutant human tau [29], linking tau hyperphosphorylation to RGC degeneration. Further research discovered increased phosphorylation of tau at serine 396 (S396) and S404, threonine 205 (T205) and T231, which may impair axonal and dendritic transport of mRNA transcripts and translational machinery, such as Golgi, mitochondria, and rough endoplasmic reticulum (RER), into distal synapses through disrupting microtubule tracks in RGCs [30, 31], thereby impairing local protein synthesis capacity. Hence, a mild retinal tauopathy has been proposed as a new pathophysiological model for DR [28]. Furthermore, rescuing of synaptic loss via knock-down of tau in the retina before RGC apoptosis occurs appears to have widespread beneficial effects on the overall health of RGCs, improving visual functions impaired by diabetes.

In sharp contrast to the structural and transport disruption caused by tau hyperphosphorylation in neurons, abnormal phosphorylation within the retinal vasculature operates as a dynamic signaling switch that drives barrier breakdown and proliferation. Prolonged hyperglycemia and retinal hypoxia lead to the excessive secretion of vascular endothelial growth factor (VEGF). Then, VEGF activates intracellular signaling by binding to VEGF receptor (VEGFR), regulating angiogenesis and vascular permeability, and serves as the core driving force of pathological angiogenesis and macular edema [32]. Currently, two receptor tyrosine kinase receptors (VEGFR1 and VEGFR2) have been identified for VEGF, with VEGFR2 primarily mediating its biological function [32]. VEGF binding to VEGFR2 triggers its tyrosine auto-phosphorylation [33, 34], which then recruits and activates key adaptor proteins. These contrasting paradigms demonstrate that the downstream effects of phosphorylation in DR manifest uniquely depending on the host cell type, acting as a chronic degenerative brake on microtubule stability in RGCs while functioning as an acute pro-angiogenic accelerator in endothelial cells.

### Glycosylation

As one of the most abundant and diverse type of PTMs, glycosylation refers to the addition of glycan moieties to proteins [43]. Glycans are crucial for cellular responses to environmental stimuli, as well as for cellular growth and differentiation [44], with specific changes in glycan composition associated with various diseases [43]. Eukaryotic protein glycosylation mainly occurs through two types: N-linked (N-glycosylation) and O-linked (O-glycosylation). It is worth mentioning that O-linked N-acetylglucosamine modification (O-GlcNAcylation) is a unique form of O-glycosylation where O-GlcNAc is added to serine (Ser) and threonine (Thr) residues of proteins in the nucleus, cytoplasm, and mitochondria [45, 46]. Research has demonstrated that O-GlcNAcylation modulates protein functions in various ways, including cellular localization, protein stability, and protein-protein interactions [47].

Growing evidence indicates that O-GlcNAcylation is essential in DR, with changes in its modification levels potentially regulating the fate of RGCs, Müller cells, astrocytes, retinal endothelial cells, and pericytes under high glucose conditions [48, 49]. In retinal endothelial cells, O*-*GlcNAc elevation in both DR patients and mouse models, promoting endothelial cells activation and angiogenesis [50]. Specifically, O-GlcNAcylation could regulate angiogenesis by targeting the Hippo-Yes-associated protein (YAP) /Transcriptional co-activator with PDZ-binding motif (TAZ) pathway [50]. Moreover, O-GlcNAcylation is enhanced by upregulating the expression of glutamine-fructose-6-phosphate aminotransferase 2 (GFAT2), which increases the flux from the glucosamine biosynthetic pathway and forms uridine 5′-diphosphate-N-acetylglucosamine (the substrate for PTM). This disrupts retinal endothelial barrier integrity, and promotes monocyte transendothelial migration [51]. In contrast to these endothelial hyper-permeability and migratory responses, elevated O-GlcNAcylation within the neural retina governs altogether different cellular fates. Another study reported RGCs loss with increased O-GlcNAcylation of Nuclear Factor kappa-light-chain-enhancer of activated B cells (NF-κB) p65 subunit in a diabetic mouse model [52]. Based on this preliminary research and the known link between impaired O-GlcNAcylation and neurodegenerative diseases, it can be inferred that elevated O-GlcNAcylation contributes to glucose toxicity in retinal neurons. However, these findings reveal that current literature remains predominantly descriptive without thoroughly demonstrating direct causality between elevated O-GlcNAcylation and pathological cellular fates.

In addition, O-GlcNAcylation and phosphorylation are known to engage in extensive crosstalk with each other, as both can modify Ser and Thr residues of proteins [53]. In fact, these two PTMs are indeed correlated in DR. In high-glucose-treated retinal endothelial cells, Lei et al. found that Thr383 as an O-GlcNAc site on YAP, which prevents phosphorylation at Ser397, stabilizing and activating YAP. This process promotes vascular dysfunction by stimulating pro-angiogenic and glucose metabolism transcriptional programs [50]. Occludin, a transmembrane component of tight junctions, showed reduced expression in DR, indicating a decline in blood-retinal barrier (BRB) function [54]. Inhibiting O-GlcNAcylation can increase expression of occludin, thus protecting BRB. In other diseases, researchers have found that increased serine/threonine phosphorylation of occludin enhances barrier function [35]. Suppressing glycosylated occludin expression can enhance its phosphorylation, thereby increasing the barrier function of tight junctions [36]. This seems to be consistent with the changes found in the retinas of diabetic rats. Liu et al. revealed connexin43 was modified by O-GlcNAcylation and phosphorylation simultaneously, and enhanced O-GlcNAcylation can downregulate the expression of zonula occluden-1 (ZO-1) and occludin by significantly reducing connexin43 phosphorylation, leading to BRB disruption and eventually causing vessel leakage [37]. These findings underscore that the dynamic O-GlcNAcylation/phosphorylation interplay acts as a molecular switch governing endothelial tight junction integrity. Consequently, targeting this specific competitive crosstalk, rather than single isolated modifications, presents a highly promising therapeutic avenue for mitigating blood-retinal barrier breakdown in DR.

In summary, further identification of O-GlcNAcylation targets and analysis of how O-GlcNAc modification affects their function will aid in understanding the molecular characteristics of DR and provide new insights into various disease-associated pathways, including apoptosis, inflammation, and metabolism.

### Acetylation

Acetylation involves the transfer of the acetyl group from acetyl-CoA to lysine or other amino acid residues on target proteins, which could regulate signal transduction and gene transcription [58]. This reversible process primarily regulated by lysine acetyltransferases (KATs, such as the MYST, GNAT, and CBP/p300 families) and lysine deacetylases (KDACs, including histone deacetylases (HDACs) and the sirtuin family) [59]. Recent studies have highlighted that non-histone acetylation could primarily affect gene transcription, DNA repair, protein folding, cell division, signal transduction, and autophagy [60]. Consequently, any disturbance in the delicate equilibrium between KAT and KDAC activities under metabolic stress can induce widespread proteomic instability, serving as an initiator for diabetic retinal insults.

Under hyperglycemic conditions, elevated acetylation of CBP/p300 stimulate NF-κB to promote inflammation [61]. Various isoforms of HDAC also contribute to diabetic vascular complications by regulating different molecular pathways [62]. For instance, HDAC6 level is significantly upregulated in both the retinas of patients with DR and corresponding experimental models [63], whereas its pharmacological inhibition via tubastatin A effectively mitigates retinal neurovascular injury [63]. Conversely, while the expression of class I HDACs (HDAC1/2/3/8) increases in experimental DR, certain class II isoforms (HDAC4/5) paradoxically decrease [64–66]. This structural divergence indicated that different HDAC isoforms play selective, non-overlapping roles in coordinating retinal epigenetic modifications and microvascular damage.

Beyond global alterations in enzyme expression, these shifts target specific pathogenic loci. Mounting evidence indicates that thioredoxin-interacting protein (TXNIP), a proinflammatory and proapoptotic factor, plays a causative role in the development of diabetes [67]. In the retinas of diabetic rats, sustained histone acetylation at the *TXNIP* promoter region directly accelerates its transcription, thereby exacerbating local inflammation and cellular death [68]. This mechanism demonstrates how metabolic stress-induced chromatin remodeling directly dictates the persistent activation of detrimental genes, reinforcing the notion that histone acetylation leads to sustains chronic retinal injury.

In contrast to the hyperactivation of classical HDACs that drive injury, the sirtuin family of NAD^+^-dependent deacetylases, which acts as a key metabolic counter-regulatory system, is markedly suppressed during DR progression. SIRT1, deacetylates p65 subunit of NF-κB on the K310 site [69], plays a therapeutic role in retinal endothelial cells, retinal capillary cell and RGC [70–73], thus maintaining retinal vascular and neuronal homeostasis. However, its activity is severely compromised in the diabetic retina due to the decrease of the intracellular NAD^+^ under persistent hyperglycemia [74]. Notably, SIRT1 could reverse AGE-induced vascular abnormalities and prevents diabetes-induced retinal damage by attenuating the expression of gp300, EDN1, and TGF-β1 [75–77]. In parallel, SIRT3, a potent mitochondrial deacetylase, is closely associated with the acetylation level of mitochondrial proteins [78]. And it also can enhance antioxidant activity and improve insulin resistance and ER dysfunction induced by high glucose [79, 80]. Depletion of SIRT3 accelerates NAD^+^ exhaustion and exacerbates DR pathology in streptozotocin-induced (STZ) animal models [81]. In addition, SIRT6, which deacetylates H3K9Ac and H3K56Ac, is downregulated in the retina of diabetic mice [82]. Taken together, the widespread suppression of the sirtuin family represents a profound breakdown of cellular defense mechanisms in DR. This metabolic failure not only leaves the retina vulnerable to mitochondrial and ER stress, but also highlights the therapeutic potential of restoring sirtuin activity to counteract glucose-induced metabolic memory.

### Methylation

Protein methylation is a common PTM occurs in both histones and non-histones [85], mediated by protein methyltransferases (PMTs) that add methyl groups (CH₃) to the N-terminus of proteins [86]. Depending on the substrate involved, PMTs can be classified into categories such as protein arginine methyltransferases (PRMTs) and protein lysine methyltransferases (PKMTs) [87, 88]. Protein methylation is essential for various cellular processes, including glucose and lipid metabolism, by modulating the function of target proteins [89, 90].

In the diabetic ocular microenvironment, aberrant methylation shifts alter the post-translational landscape of core survival proteins, driving them toward pathological inactivation or hyperactivation. Studies indicate that hypermethylation, through the addition of methyl groups, leads to transcription inactivation, resulting in the accumulation of toxic proteins and ultimately causing neuronal cell death [91]. PRMT4, a methyltransferase that predominantly methylates H3R17, is elevated in the retinal pigment epithelial (RPE) layer of diabetic rats, promoting oxidative stress and inducing cell death [92]. Similarly, monomethylation of histone H3 lysine 4 (H3K4me1), and di- and tri-methylation of histone H3 lysine 9 (H3K9me2/me3) promote endothelial dysfunction in diabetic vessels by inducing reactive oxygen species (ROS) [93]. Moreover, mono- and di-methylation states of histone H4 lysine 20 (H4K20me1/me2) are found to be upregulated in the Müller cells of diabetic rats, promoting development of DR [94]. These site-specific PTM events selectively alter the conformational stability of key metabolic and signaling enzymes, locking retinal endothelial cells and neurons into a chronic pro-oxidant state that directly perpetuates vascular regression and neurodegeneration.

### Ubiquitination

Protein ubiquitination is a highly regulated process involving an enzymatic cascade that attaches ubiquitin to substrate proteins, which is a reversable reaction mediated by ubiquitin ligases and deubiquitinating enzymes (DUB) [96]. The process begins with ubiquitin-activating enzymes (E1), with only two types [97], which catalyzes ATP-dependent ubiquitin activation to form a high-energy thioester bond. The activated ubiquitin is then transferred to ubiquitin-conjugating enzyme (E2), of which there are approximately 30–40 types [97]. Ubiquitin ligase (E3), with nearly 1,000 types [98, 99], which modifies proteins through protein-based recognition motifs or structural features, conferring specificity to the ubiquitination process [100].

The aberrant expression and activation of specific E3 ubiquitin ligases directly disrupt homeostatic signaling across distinct cellular layers in the diabetic retina. For example, Parkin, an E3 ubiquitin ligase, induces proteasomal degradation of its substrates through polyubiquitination and mitophagy [101, 102]. In RPE cells, the downregulated expression of Parkin inhibits the ubiquitination and degradation of synaptophysin 4 (SYT4), which promotes abnormal cellular glucose and exacerbates the progression of DR [103]. Another E3 ubiquitin ligase HERC3 is speculated to specifically and uniquely ubiquitinate a key protein or set of proteins within photoreceptors (especially rod photoreceptors) in DR [104]. In addition, the E3-ligase tripartite motif (TRIM) family exerts crucial effects on a variety of biological activities in DR onsetting. For instance, high glucose dramatically upregulates TRIM46 protein expression in a dose-dependent way. Overexpressed TRIM46 can interact with inhibitor of nuclear factor kappa B alpha (IκBα), promoting its ubiquitination and degradation, thereby leading to cellular growth inhibition, cell cycle arrest, hyper permeability, and proinflammatory cytokine secretion in human retinal endothelial cells (HRCEC), which are closely related to the occurrence of DR [105]. Similarly, TRIM25 exacerbates oxidative stress and epithelial cell apoptosis through ubiquitination of DNA repair enzymes XRCC5 and Ku80 [106, 107]. In contrast, TRIM40 could enhance K48-linked polyubiquitination of DAB1, promoting DAB1 degradation, thereby alleviating high glucose-induced inflammatory responses and also exerting a protective effect on STZ-induced DR phenotypes in mouse models [108]. These site-specific actions demonstrate that E3 ligases function as molecular switches within individual retinal cell lineages, where the selective turnover of regulatory proteins can either unleash localized microvascular inflammation or compromise outer retinal structural survival.

Counteracting these ligases, DUBs remove ubiquitin chains from substrates to rescue them from degradation, and based on the structure of their catalytic domains, they can be divided into eight subfamilies, the largest of which is the ubiquitin-specific proteasome (USP) [109]. For instance, in RPE cells, USP48 overexpression deubiquitinate SLC1A5 to promote cell proliferation, and suppress cell apoptosis, inflammation, ferroptosis, and oxidative stress, thereby inhibiting the progression of DR [110]. In contrast, USP5/14/25 accelerate DR pathology through substrate stabilization. USP5 could repress cell proliferation and aggravate high glucose-induced HRPE cell damage by deubiquitinating Roundabout 4 (ROBO4) protein [96]. Within Müller cells, USP14 reduces IκBα ubiquitination, which fuels IκBα/ and NF-κB activation, ultimately promoting activation of inflammatory responses in these glia [111]. USP25 triggers the Rho-associated coiled-coil containing protein kinase (ROCK) signaling pathway, leading to NF-κB phosphorylation, enhanced glial cell activation, increased inflammatory factor secretion, and enhanced retinal inflammatory response [112]. The functional divergence among these USPs reinforces that deubiquitinating activities in DR are strictly substrate- and cell-type-dependent, where the pathological stabilization of signaling components within glial and epithelial support networks can actively transform them into chronic drivers of neuroinflammation.

Additionally, in diabetic retina, hyperglycemia cause the upregulation of ubiquitin–proteasome system (UPS) due to increased ROS. As a consequence, glucose transporter 1 (GLUT1) undergoes mono- or di-ubiquitinated, tagging it for endocytosis and subsequent lysosomal degradation, which reduces glucose transport into retinal endothelial cells [113]. This altered protein trafficking is accompanied by the hyperphosphorylation of Cx43, a connexin subtype expressed in the endothelium of blood vessels, which triggers its degradation in bovine retinal endothelial cells through a proteasome-dependent mechanism. Furthermore, the peptide hormone angiotensin II (Ang II), which is elevated in DR, may trigger an upregulation of ubiquitin-conjugated synaptophysin expression through UPS-mediated mechanisms, subsequently impairing synaptic activity, adversely affecting the survival of RGC and inner retinal cells and visual function [114]. Collectively, these substrate-specific ubiquitination events translate systemic metabolic perturbations into the literal dismantling of physical vascular barriers, metabolic transporters, and neuro-synaptic connections, illustrating how global UPS hyperactivation drives the progressive anatomical and functional decline characteristic of advanced DR.

### SUMOylation

SUMOylation is a type of PTMs similar to ubiquitination, catalyzing the covalent binding of small ubiquitin-like modifier (SUMO) proteins (SUMO1/2/3) to lysine residues in target proteins. This process is regulated by specific SUMO E1 activating enzyme, SUMO E2 conjugating enzyme, and SUMO E3 ligases. Currently, many studies have found that an imbalance between SUMOylation and deSUMOylation occurs in various diseases [115, 116]. An imbalance between the opposing forces of SUMOylation and deSUMOylation disrupts critical cellular sensing networks, playing a foundational role in the systemic dysregulation of glucose and lipid homeostasis.

Improper SUMOylation of proteins is directly related to diabetes [117]. In the early stages of diabetes, enhanced SUMOylation of glial maturation factor beta (GMFB) can be detected in RPE cells, a modification that drives a robust local inflammatory response [118]. This outer retinal stress occurs in tandem with a distinct disruption of the deSUMOylation machinery in the inner retinal vasculature. In the retina of diabetic mouse, levels of SUMO specific peptidase 1 (SENP1) are markedly reduced, inhibiting SUMO2/3 modification and subsequently repressing the expression of RUNX family transcription factor 1 (RUNX1). This phenomenon would promote pathological proliferation of mouse retinal microvascular endothelial cells (mRMEC), thus exacerbating DR symptoms [119]. Differently, SUMO1 possesses a distinct protective capacity, specifically, it may inhibit Nox1-driven ROS production in human retinal epithelial cell (HREC) via SUMOylation-dependent UBC9-mediated intracellular transport [120]. In addition, SENP3 expression was increased in mRMEC with diabetes, leading to Drp1 deSUMOylation, which disrupted mitochondrial dynamics and triggers endothelial cell apoptosis [121]. This functional compartmentalization and the stark divergence between individual SUMO isoforms underscore that DR progression is not driven by a generalized shifting of post-translational metabolic traits, but rather by highly site-specific enzymatic imbalances. Consequently, mapping these precise cell-type-specific SUMOylation signatures across the retinal layers will be vital for shifting therapeutic interventions from broad-spectrum enzyme modulation toward targeted molecular stabilization.

## The role of PTMs in different signaling cascades in DR

PTMs are essential in regulating several biological signaling pathways, many of which are critically implicated in the underlying mechanisms of DR. This section will explore the signaling pathways associated with DR and examine how various PTMs contribute to their modulation.

### PI3K/Akt signaling pathway

The phosphoinositide 3-kinase (PI3K)-Akt signaling pathway is a critical intracellular signaling cascade that regulates cell survival, proliferation, metabolism, and migration [123, 124]. Activation of this pathway is crucial to the pathophysiology of DR [125], as it acts as a convergence point for various growth factors, pro-inflammatory mediators, and downstream substrates that regulate cellular survival processes vital for the initiation and progression of angiogenic cascades [126]. In the PI3K-Akt signaling pathway, ligands (such as insulin, insulin-like growth factor-1, VEGF, inflammatory cytokines, AGEs, etc.) bind to their specific cell surface receptors, resulting in receptor activation. This subsequently leads to the recruitment and activation of the lipid kinase PI3K, which then phosphorylates the membrane lipid phosphatidylinositol 4,5-bisphosphate (PIP2), converting it into phosphatidylinositol 3,4,5-trisphosphate (PIP3). As a second messenger, PIP3 mediates further intracellular signaling [127]. In addition, PIP3 recruits Akt to the plasma membrane, binding through the pleckstrin homology domain of Akt. This complex induces a conformational change in Akt, enabling its phosphorylation at T308 by phosphoinositide-dependent kinase 1 (PDK1) [128] and at S473 by mammalian target of rapamycin complex 2 (mTORC2), leading to the complete activation of Akt [129]. Importantly, under diabetic stress, downstream pathological consequences of this signaling cascade are strictly dictated by the specific retinal cell type in which it operates.

There are three subtypes of Akt (Akt1/2/3), among which Akt2 has been shown to play a role in diabetes [130]. In DR, Akt2 activity is increased, driven by ROS through the PI3K/PDK1 signaling pathway. Furthermore, in the RPE of DR mice, Akt1 and Akt2 are mutually regulated. Specifically, RPE cell-specific knockout of Akt2 adaptively upregulates phospho-Akt1, thereby inhibiting vascular damage, inflammatory cytokine release, and alleviating diabetes-induced retinal abnormalities [131]. In addition, endothelial cells treated with high glucose exhibit an increase in tyrosine nitration of the p85 subunit of PI3K, which inhibits its interaction with p110 subunit, thereby weakening PI3K/Akt kinase activity. This suppression of survival signals accelerates endothelial cell death [132].

**Fig. 4:**
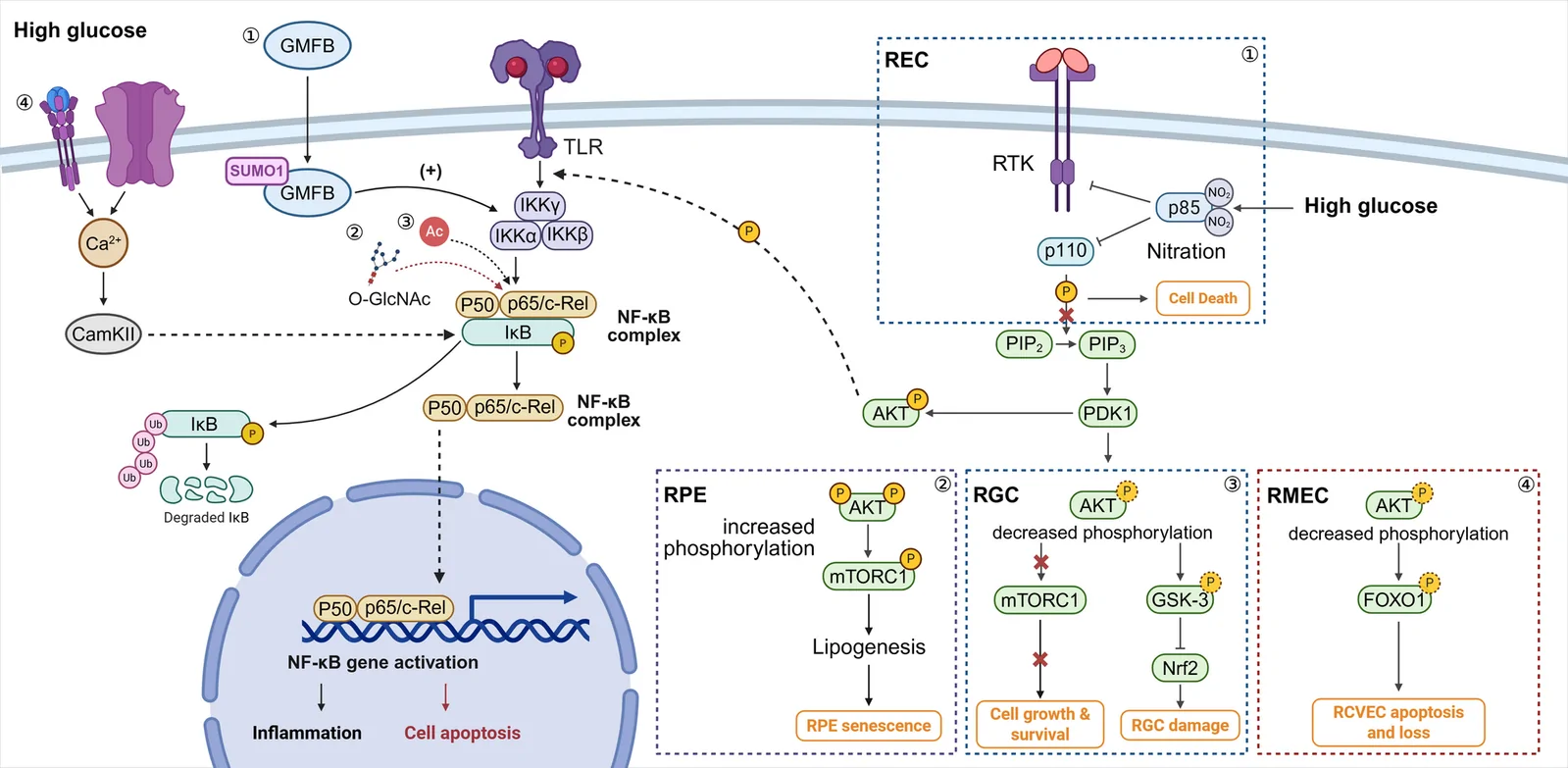
Implementations of PTMs on PI3K/Akt and NF-κB signaling pathway. The PI3K/Akt signaling pathway is a critical intracellular signaling cascade. In the PI3K/Akt signaling pathway, ligands, bind to their specific cell surface receptors, resulting in receptor activation. This subsequently leads to the recruitment and activation of the lipid kinase PI3K. Activated PI3K then phosphorylates the membrane lipid phosphatidylinositol 4,5-bisphosphate (PIP2), converting it into phosphatidylinositol (3,4,5)-trisphosphate (PIP3), which acts as a second messenger to mediate the remaining intracellular signaling. In addition, PIP3 recruits Akt to the plasma membrane and docks it through the pleckstrin homology domain of Akt. In retinal endothelial cells, high glucose exhibits an increase in tyrosine nitration of the p85 subunit of PI3K, which inhibits its interaction with p110 subunit, thereby weakening PI3K/Akt kinase activity. High glucose could induce phosphorylation of Akt and mTORC1, contributing to ROS-mediated cellular senescence in RPE. In the early stage of DR, decreased Akt phosphorylation promote the increased activity of GSK-3 and inhibit expression of Nrf2, thus leading to RGC damage. High glucose inhibits Akt to activate the function of FOXO by dephosphorylation FOXO, promoting RMECs apoptosis and loss. In addition, AKT, as an enzyme that phosphorylates IκB kinase, could lead to the activation of the IKK complex and subsequent phosphorylation of IκBα. The phosphorylated IκBα is then ubiquitinated and degraded by the proteasome, thereby releasing the p65/p50 heterodimer of NF-κB. Then, p65/p50 heterodimer translocate into nuclear, and binds to κB sites on the promoters of target genes, thereby inducing the expression of inflammatory cytokines. SUMOylation of GMFB could lead to upregulation of NF-κB, contributing to DR inflammation. Increased O-GlcNAcylation of the p65 subunit of NF-κB enhances its transcriptional activity and promotes its nuclear translocation. Further, increased NF-κB p65 acetylation was also found in retinal endothelial cells treated with high glucose, causing inflammation and metabolic disorders in the retina. In addition, there also exists an early and atypical NF-κB signaling pathway regulated by a CamKII-proteasome axis in experimental model of DR. GMFB, glial maturation factor beta; TLR, Toll-like receptor; CamKII, calcium-dependent calmodulin kinase II; REC, retinal endothelia cells; RTK, Receptor Tyrosine Kinase; RPE, retinal pigment epithelia; RGC, retinal ganglion cells; GSK-3; glucogen synthase kinase-3; Nrf2, nuclear factor erythroid 2-related factor 2; RMEC, retinal microvascular endothelial cells; FOXO1, Forkhead box O1

mTOR is an atypical serine/threonine protein kinase and a substrate of PI3K/Akt signaling. PI3K/Akt/mTOR plays a crucial role in oxidative stress-induced DR. High glucose could induce phosphorylation of Akt and mTORC1, contributing to ROS-mediated cellular senescence [133]. Therefore, inhibiting the phosphorylation of this pathway can suppress hyperglycemia-induced inflammatory damage in human RPE cells. However, Yan et al. found that treatment of RGC-5 cells with palmitic acid decrease the phosphorylation of Akt, promoting apoptosis of RGC-5 cells [134]. Thus, the specific role of Akt in different retinal cells deserve further investigation. The proapoptotic transcription factor FOXO is also a downstream target of Akt. Interestingly, activated Akt phosphorylates FOXO to inhibit its function, thereby promoting cell survival, growth and proliferation [135]. Thus, high glucose inhibits Akt to activate FOXO, promoting rat retinal microvascular endothelial cells (rRMECs) apoptosis and loss [136]. In addition, Akt could inactivate GSK-3β by phosphorylating it, which in turn activates nuclear factor erythroid 2-related factor 2 (Nrf2) [137]. In the early stage of DR, decreased Akt phosphorylation promote the increased activity of GSK-3 and inhibit expression of Nrf2, thus leading to RGC damage [28]. In addition, AKT, as an enzyme that phosphorylates IκB kinase, is the upstream/regulator that controls NF-κB activation. In the retina of diabetic rats, the expression of PI3K, phosphor-Akt, and the downstream, phosphor-NF-κB, are all upregulated [138]. In summary, the seemingly contradictory dual roles of the PI3K/Akt pathway documented in DR literature are largely a reflection of cell-type-specific responses. On one hand, diabetic stress triggers its hyperactivation to drive inflammation and senescence in RPE cells. On the other hand, it suppresses this survival signaling to execute apoptosis in both microvascular endothelial cells and retinal ganglion cells. Therefore, Therefore, future research should focus on these cell-type-specific variations to develop targeted therapies. Understanding how the PI3K/Akt pathway functions in individual retinal cells is essential to design precise treatments that protect both neurons and vessels without harming other healthy tissues (Fig. 4).

### MAPK signaling pathway

Mitogen- activated protein kinase (MAPK) signaling pathway regulates cell proliferation, survival, differentiation, apoptosis, immune response, and stress response [139, 140]. Three key MAPK families—p38 MAPK, c-Jun N-terminal kinase (JNK), and extracellular signal regulated kinase 1/2 (ERK 1/2)—are involved in intracellular signal transduction [141]. Each MAPK family is activated through a cascading process of three mutually interacting specific enzymes, namely MAP3K, MAP2K, and MAPK [142]. First, when the extracellular ligand binds to the cell receptor (e.g., VEGF binding to VEGFR could induce activation of MAPK signaling pathways [143]), MAP3K is activated through phosphorylation. Then phosphorylated MAP3K activates MAP2K, and the activated MAP2K subsequently initiates MAPK by phosphorylating MAPK at Thr and Tyr residues [142]. The activated MAPK subsequently phosphorylates various downstream substrates at Ser and Thr residues, thereby regulating a range of cellular processes [144].

The MAPK families are closely associated with DR. From a sub-family perspective, the biological consequences of MAPK activation cannot be viewed as a uniform pathogenic event, as individual members wield opposing influences on endothelial fate. In diabetic mouse models, VEGF-induced VEGFR2 autophosphorylation leads to phosphorylation of MAPK/ERK and MAPK/JNK, promoting the formation of pathological blood vessels and exacerbating DR [143]. Conversely, the levels of VEGF can also be regulated by phosphorylation of above MAPK families [145, 146]. Specifically, succinate levels are elevated in the retina of diabetic rats, thus activating its receptor G-protein-coupled receptor 91 (GPR91), then inducing the phosphorylation of ERK1/2 and JNK, which upregulates COX-2 and PGE_2_ expression and mediates VEGF secretion [147]. Excessive production of VEGF unsettles the BRB, leading to vascular leakage and neovascularization [148]. In sharp contrast to these pro-permeability cascades, ERK5, as the newly identified member of the MAPK family, primarily exerts its function through phosphorylation of the Thr-Glu-Tyr (TEY) activation motif in N-terminal, playing a crucial role in maintaining endothelial function and cell integrity. In early stage, high glucose could lead to increased ERK5 phosphorylation in retina of diabetic rats to inhibit partial fibronectin production to prevent endothelial injury; however, in the late stage, ERK5 phosphorylation decreased, thus leading to fibronectin accumulation [149]. Other studies have shown that high glucose-induced AGEs and H2O2 promote ERK5 SUMOylation, impairing its transcriptional activity and resulting in diabetes-mediated endothelial dysfunction [150]. This contrast within the same cellular compartment reveals that the vascular outcome of DR is determined by a balance between different MAPK branches. While ERK1/2 and JNK drive the early angiogenic and inflammatory responses, the simultaneous impairment of the protective ERK5 branch further accelerates the breakdown of the endothelial barrier. Therefore, the progression of vascular leakage is not just caused by the hyperactivation of damaging signals, but also by the parallel loss of endogenous defense mechanisms within the endothelium.

**Fig. 5:**
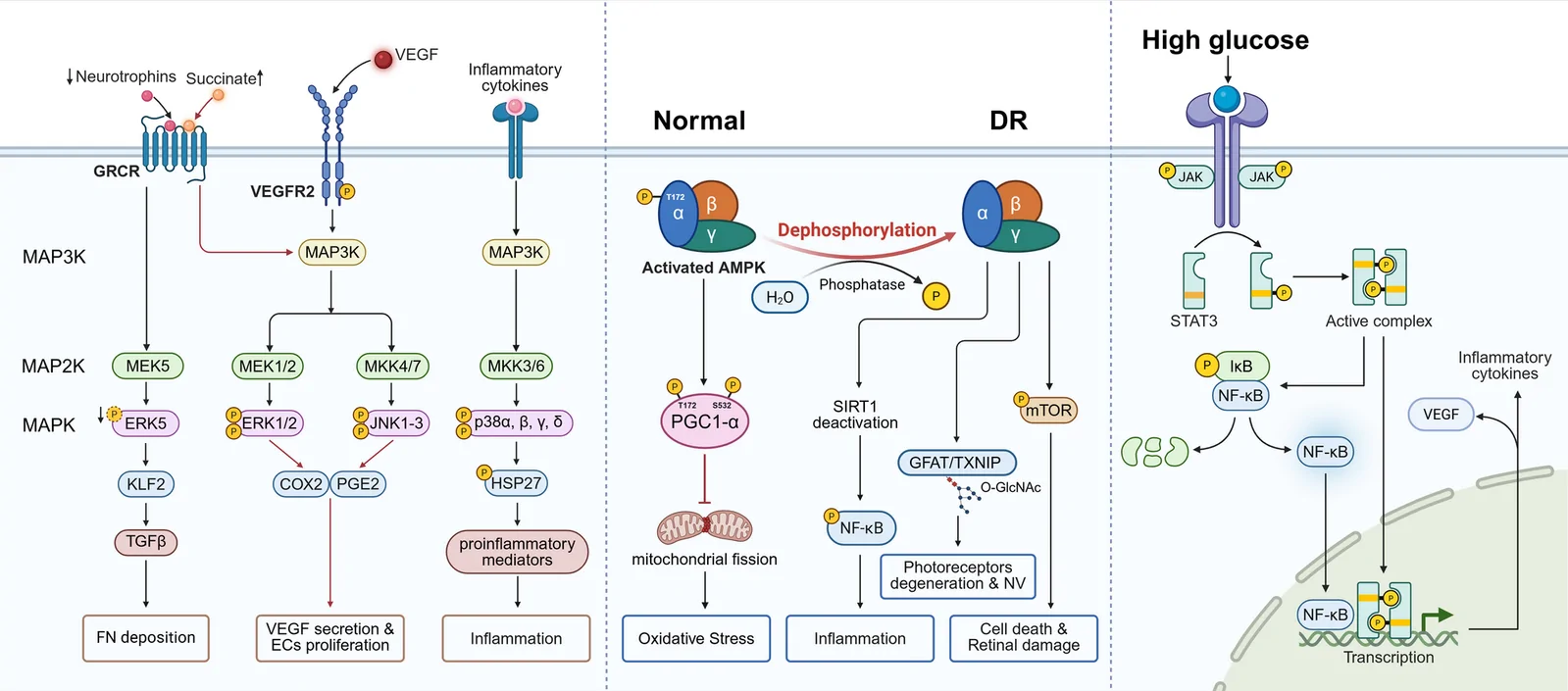
Implementations of PTMs on MAPK cascade, and AMPK and JAK/STAT signaling pathway. MAPK signaling pathway is a biological signaling pathway that includes three key MAPK families (p38 MAPK, JNK and ERK1/2/5). Each MAPK family is activated through a sequential cascade of three mutually interacting specific enzymes, namely MAP3K, MAP2K, and MAPK. First, when the extracellular ligand binds to the cell receptor, MAP3K is activated through phosphorylation. Then phosphorylated MAP3K activates MAP2K, and the activated MAP2K subsequently initiates MAPK by phosphorylating MAPK at threonine and tyrosine residues. In diabetic mouse models, VEGF-induced VEGFR2 autophosphorylation leads to phosphorylation of MAPK/ERK and MAPK/JNK, promoting the formation of pathological blood vessels and secretion of VEGF. Succinate levels are elevated in the retina of diabetic rats, thus activating its receptor in GPR91, then inducing the phosphorylation of ERK1/2 and JNK, which upregulates COX-2 and PGE_2_ expression and mediates VEGF secretion. In the late stage of DR, ERK5 phosphorylation decreased, leading to fibronectin accumulation. Moreover, the phosphorylation of p38 is increased in retina of diabetic rats, leading to diabetes-induced increase in HSP27 phosphorylation, which promote the inflammation. AMPK is composed of a single catalytic α-subunit, regulatory β-subunit, and AMP-binding γ-subunit. In normal condition, the activation of AMPK causes PGC-1α to be phosphorylated at the T172 and S538 residues, suppressing mitochondrial fission, which protects RPE cells in diabetic mouse against oxidative stress. Compared with the normal retina, AMPK activity in the diabetic retina significantly is declined, specifically manifested as AMPK dephosphorylation. In addition, in the retina of STZ-induced type 1 diabetic rats, decreased phosphorylation of AMPK would lead to increased phosphorylation of mTOR, causing autophagy pathway dysregulation, thus proceed retinal damage and cell apoptosis. The induction of diabetes in mice also could lead to retinal SIRT1 deactivation and AMPK deactivation, thus leading to NF-κB-induced inflammation. Moreover, during DR, AMPK inactivation could make O-GlcNAc modification and diabetes-induced photoreceptor cell degeneration increase. The JAK/STAT pathway is activated when an extracellular ligand binds to its receptor on the cell surface, leading to the phosphorylation of associated JAKs. Then, JAKs phosphorylate specific tyrosine residues on the receptor, creating docking sites for STAT proteins. STATs are then recruited to the receptor, where they are phosphorylated by JAKs on tyrosine residues. high glucose activates the JAK/STAT motion of vascular endothelial cells and further promote production of VEGF by phosphorylating JAK2 and STAT3. Further, retinal STAT3/NF-κB in STZ-induced diabetic rats is upregulated, thus leading to increased production of inflammatory cytokines. GPCR, G-protein-coupled receptor; VEGF, vascular endothelia growth factor; VEGFR2, vascular endothelia growth factor receptor 2; MAPK, Mitogen- activated protein kinase; ERK, extracellular signal-regulated kinase; JNK, c-Jun N-terminal kinase; KLF2, Kruppel-like Factor 2; COX2, cyclooxygenase-2; PGE2, Prostaglandin E2; HSP27, heat shock protein 27; TGFβ, transforming growth factor β; FN, fibronectin; ECs, endothelial cells; AMPK, adenosine monophosphate-activated protein kinase; PGC-1α, proliferator-activated receptor-gamma coactivator-1α; GFAT, glutamine-fructose-6-phosphate aminotransferase; TXNIP, thioredoxin-interacting protein; mTOR, mammalian target of rapamycin; NV, neovascularization; JAK, Janus kinase; STAT3, signal transduction and activators of transcription 3

p38 activation is crucial in the long-term vascular pathology of DR. For example, the phosphorylation level of p38 correlates with the viability of RF/6A cells in both high glucose conditions and the STZ diabetic rat [151]. Retinal endothelial cells treated with high glucose would lead to increases in phosphorylation of p38 MAPK, which drives pro-apoptotic effects and accelerates cell death [132]. In vivo, the phosphorylation of p38 is increased in retina of diabetic rats, leading to diabetes-induced increase in phosphorylation of heat shock protein 27 (HSP27), which promote the development of vascular disease [152]. Moreover, the phosphorylation of p38 MAPK could also enhances and regulates the expression of pro-angiogenesis factors, such as VEGF [153, 154]. However, its role in regulating VEGF in DR still remain unclear, thus waiting for further exploration. Clarifying this potential link will be essential to fully understand the exact boundaries of p38 pathological functions during DR progression (Fig. 5).

### AMPK signaling pathway

Adenosine monophosphate-activated protein kinase (AMPK) is an evolutionarily conserved master kinase regulating multiple cell functions [155]. It is composed of a catalytic α-subunit, a regulatory β-subunit, and an AMP-binding γ-subunit [156]. Each subunit exists in multiple isoforms (α₁-α₂, β₁-β₂, γ₁-γ₂-γ₃), which are encoded by different genes, resulting in multiple heterotrimeric combinations [157]. The α subunit of AMPK is phosphorylated at T172 by various kinases, such as Calmodulin dependent protein kinase kinase-β (CaMKKβ) and liver kinase B1 (LKB1), resulting in conformational changes in AMPK [158]. Phosphorylation of AMPK enhances its activity, which then regulates downstream substrates. During ATP depletion conditions, myristoylation of the AMPK β subunit facilitates allosteric binding between AMP/AMPK and phosphorylation of the α subunit at the T172 [159]. Similarly, SUMOylation of the AMPKβ2 subunit via SUMO2 promotes the overall activity of AMPK [160]. However, the ubiquitin ligase inhibits AMPK activity by ubiquitin-dependent proteasomal degradation of the AMPK β subunit [161]. In addition, H₂O₂-mediated oxidation of cysteine residues in the AMPK α and β subunits increases AMPK kinase activity [162]. There are also reports that H₂O₂-mediated S-glutathionylation of the AMPK α subunit at C299 and C304 promotes AMPK activation [163].

Unlike other signaling pathways that exhibit cell- or member-specific variations, the AMPK axis shows a consistently suppressed pattern in the diabetic retina, acting as a failing metabolic guardian whose inactivation triggers downstream multi-organelle dysfunction. For example, phosphorylated AMPK in mouse photoreceptor-derived (661w) cells decreased after AGEs treatment, promoting diabetes-induced photoreceptor cells degeneration [164]. And compared with the normal retina, AMPK activity in the diabetic retina significantly is declined, specifically manifested as AMPK dephosphorylation [165]. The activation of AMPK causes proliferator-activated receptor-gamma coactivator-1α (PGC-1α) to be phosphorylated at the T172 and S538 residues, suppressing mitochondrial fission and enhancing mitochondrial biogenesis, which protects RPE cells in diabetic mouse against oxidative stress [166]. And in the retina of STZ-induced type 1 diabetic rats, researchers found decreased phosphorylation of AMPK would lead to increased phosphorylation of mTOR, causing autophagy pathway dysregulation, thus proceed retinal damage and cell apoptosis [167]. Interestingly, recent in vitro data have shown that AMPK activation inhibits NF-κB-induced inflammatory responses in macrophages through the effect of SIRT1 on deacetylating NF-κB [168], which is the same mechanism observed in ARPE-19 cells treated with high glucose [169]. And the induction of diabetes in mice led to retinal SIRT1 deactivation and AMPK deactivation, thus leading to NF-κB-induced inflammation [165]. AMPK activation by its agonist could reduce O-GlcNAc modification and diabetes-induced photoreceptor cells degeneration during DR [170].

In summary, AMPK acts as a universal protective axis in the retina, and its deactivation under diabetic stress leads to a coordinated failure of mitochondrial biogenesis, autophagy, and inflammation control. Therefore, future research should focus on exploring potent AMPK activators to restore this central defense system and protect multiple retinal cell types simultaneously (Fig. 5).

### NF-κB signaling pathway

NF-κB is a highly conserved pro-inflammatory signaling pathway that is critical for maintaining neuronal homeostasis [171]. Under hyperglycemic conditions, oxidative stress is enhanced in retinal cells, leading to the excessive production of ROS and AGEs. Then, AGEs activate the RAGE signaling pathway, which leads to the activation of the IKK complex and subsequent phosphorylation of IκBα. The phosphorylated IκBα is then ubiquitinated and degraded by the proteasome, thereby releasing the p65/p50 heterodimer of NF-κB [172]. The differential binding kinetics of p65 with various importin/exportin proteins, along with the regulatory effects of PTMs, provide additional mechanisms for the modulation of NF-κB activity, ultimately determining the extent of p65 nuclear translocation [173–175]. A variety of PTMs, such as phosphorylation, O-GlcNAcylation, and acetylation, regulate the NF-κB/p65 activation and its downstream effects [176]. Thus, activated NF-κB, regulated by PTMs, binds to κB sites on the promoters of target genes, thereby inducing the expression of inflammatory cytokines, including tumor necrosis factor-alpha (TNF-α), interleukin-6 (IL-6), IL-1β, and monocyte chemoattractant protein-1 (MCP-1). These molecules, along with increased expression of cell adhesion molecules, contribute to retinal inflammation and microvascular damage in DR [177, 178].

Depending on the specific retinal cell type, NF-κB is driven by different types of PTMs to cause distinct forms of damage. In RGC, increased O-GlcNAcylation of NF-κB p65 subunit enhances its transcriptional activity and promotes its nuclear translocation, thereby leading to RGC apoptosis [179]. In retinal endothelial cells treated with high glucose, increased NF-κB acetylation was also found to cause inflammation and metabolic disorders in the retina [180]. Likewise, levels of acetylated NF-κB p65 and p53 proteins were significantly increased in high glucose conditions [169]. In RPE cells, the pathway is activated through a different modification entirely, where the SUMOylation of GMFB causes the upregulation of NF-κB and drives localized inflammation [118]. These findings highlight a complex, multi-layered regulatory network where the same core pathway is differentially driven by distinct PTM depending on the microenvironment of each retinal cell type. Thus, fully deciphering these heterogeneous PTM profiles on NF-κB is essential for designing targeted therapeutic strategies that can selectively mitigate inflammation in specific retinal cell layers.

The complexity of NF-κB signaling in DR is further compounded by alternative activation mechanisms that bypass these canonical checkpoints. Recently, Sbardella et al. has demonstrated that there exists an early and atypical NF-κB signaling pathway regulated by a calcium-dependent calmodulin kinase II (CamKII)-proteasome axis in experimental model of DR in vitro. Specifically, phosphorylation of proteasome subunit Rpt6 at S120 by CamKII stimulates the turnover of IkBα (the natural inhibitor of NF-κB p65/50 complex), independent of the phosphorylation at S32 which represents canonical NF-kB signaling [181]. These parallel mechanisms, including cell-specific modifications and alternative activation pathways, collectively maintain the chronic inflammatory state in the diabetic retina (Fig. 4).

### JAK/STAT signaling pathway

The Janus kinase (JAK)/ signal transduction and activators of transcription (STAT) pathway is a major regulator of cytokine signaling and participates in mediating processes related to angiogenesis. It comprises four non-receptor tyrosine kinases (JAKs) and seven transcription factors (STATs), which are regulated by phosphorylation on specific Ser and Tyr residues [182]. The JAK/STAT pathway is activated when an extracellular ligand, such as a cytokine, binds to its receptor on the cell surface, leading to the activation of associated JAKs. Upon ligand binding, JAKs phosphorylate specific tyrosine residues on the receptor, creating docking sites for STAT proteins. STATs are then recruited to the receptor, where they are phosphorylated by JAKs on tyrosine residues. This phosphorylation induces the dimerization of STAT proteins, which then translocate to the nucleus [183].

In the context of DR, this pathway acts as a direct driver of retinal microvascular leakage by regulating angiogenic factors. In vascular endothelial cells, high glucose activates the JAK/STAT pathway by phosphorylating JAK2 and STAT3 [184], thus leading to overproduction of VEGF [185]. This hyperactivation is consistently observed across various diabetic models. For instance, in db/db mice, albumin leakage is accompanied by elevated levels of phosphorylated JAK1 [186]. Following STZ injection to induce diabetes, the resulting increased phosphorylation of JAK and STAT3 allows activated STAT3 to bind to the VEGF promoter [187, 188]. This transcriptional upregulation of VEGF subsequently suppresses ZO-1 and occludin levels, which compromises tight junctions and elevates endothelial cell permeability [188]. The clinical relevance of this axis is highlighted by findings that increased expression of phosphorylated STAT3 is observed in the diabetic retinas, and targeting STAT3 can inhibit high glucose-induced VEGF upregulation and migration of human retinal microvascular endothelial cells [189], thus protecting BRB integrity (Fig. 5). However, while these studies consistently link the pathway to vascular leakage, the distinct functional roles and individual contributions of different JAK isoforms (such as JAK1 versus JAK2) remain to be fully clarified in vivo. Future research should focus on deciphering these subunit-specific mechanisms to develop more targeted therapeutic interventions without disrupting broader cytokine signaling.

## Mechanistic role of PTMs on cellular dysregulation process in DR

**Fig. 6:**
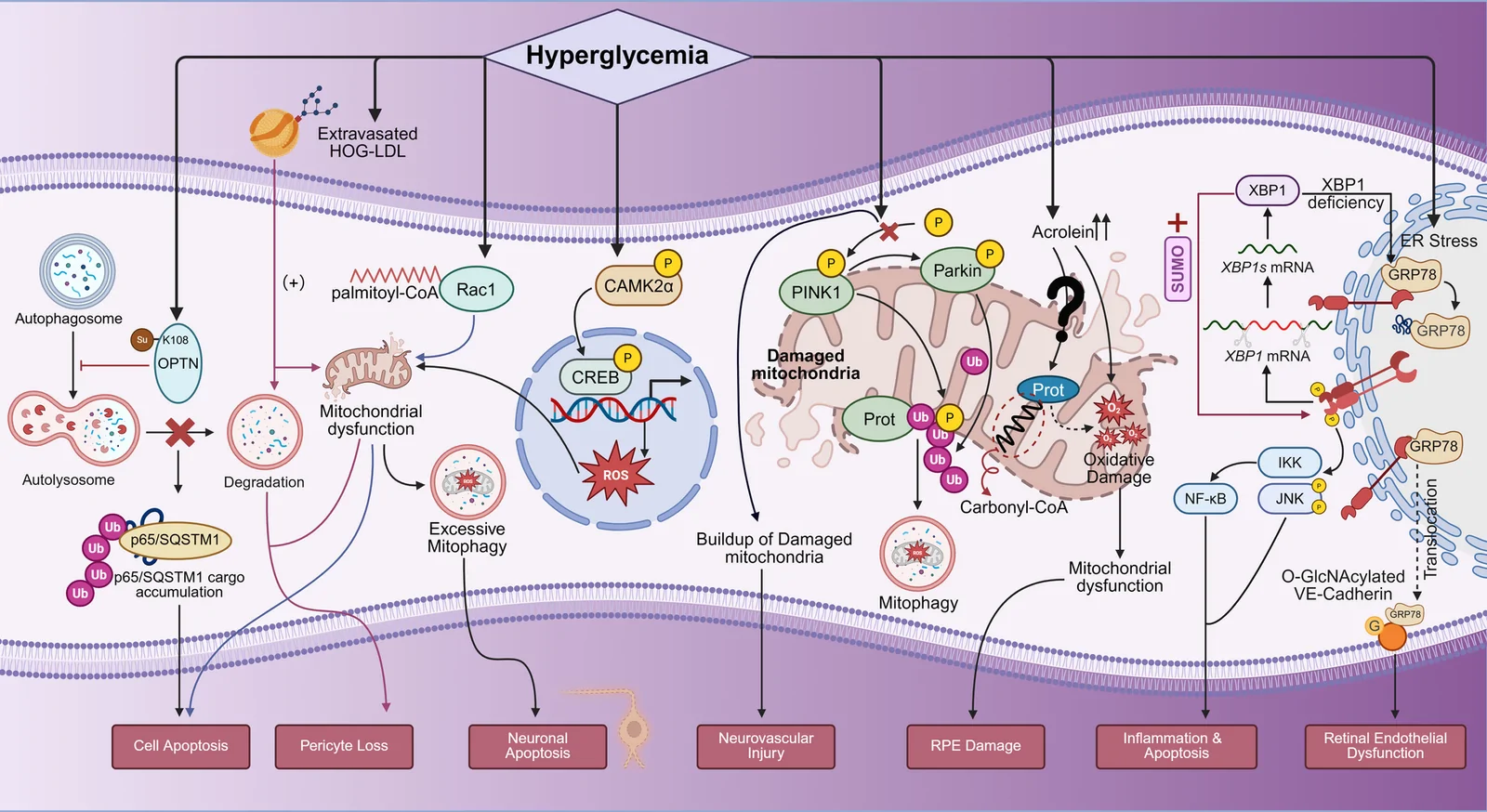
Mechanistic role of PTMs on cellular process in DR. (1) In the retina of rats with diabetes, succinylation of the K108 site of OPTN could mediate the blockade of the autophagy pathway under high glucose condition. When autophagic flux is compromised, the function of shuttling the ubiquitinated substrates to autophagy degradation through UBA domain and LC3 interacting region of p62/SQSTM1 would be destroyed, leading to p62/SQSTM1 cargo accumulate, finally resulting in neurodegenerative disorders. (2) The barrier is weakened in DR, causing glycated LDL to leakage, thus increasing autophagy and mitochondrial dysfunction, finally resulting in pericyte loss. (3) The hyperglycemic milieu promotes palmitoylation of Rac1, leading to accumulation of ROS, which damage the mitochondrial membrane, proteins, and DNA, eventually stimulating cell apoptosis. (4) In DR model, the retina tissue could express enhanced phosphorylation of CaMK2α and CREB, subsequently, increased ROS would lead to mitochondrial dysfunction and excessive mitophagy, finally leading to retinal neuronal degeneration. (5) At advanced stages of neurovascular injury in the diabetic retina, PINK1 cannot be phosphorylated and therefore cannot cause phosphorylation of ubiquitin bound to outer mitochondrial membrane protein, thus disrupting mitophagy and mitochondrial biogenesis, resulting in the buildup of damaged mitochondria, finally causing neovascular injury. (6) Hyperglycemia leads to elevated acrolein levels, which increases oxidative damage in mitochondria, resulting in RPE damage. Interestingly, elevated levels of protein carbonyl are also found in DR patients, however, it remains unclear whether the increased level of acrolein will promote oxidative stress through hydroxylated proteins in DR. (7) Upon ER stress, GRP78 dissociates from IRE1 and binds to accumulated unfolded or misfolded proteins. Then, IRE1 is activated by dimerization and autophosphorylation. Increased kinase activity of IRE1 promotes JNK and IKK activation resulting in inflammation and apoptosis. Moreover, the endoribonucease domain of IRE1 is activated resulting in an unconventional splicing of XBP1 mRNA. In diabetic mice, XBP1 deficiency resulted in elevated levels of GRP78 in retina, promoting pronounced vascular leakage. However, the spliced XBP1 can also be SUMOylated to elevate ER stress-induced apoptosis. In addition, ER stress could induce the translocation of GRP78 to the plasma membrane, which increases O-GlcNAcylation of VE-Cadherin, resulting in defective junctions leading to endothelial hyperpermeability. HOG-LDL, heavily oxidized, glycated low density lipoprotein; OPTN, Optineurin; Rac1, Ras-related C3 botulinum toxin substrate 1; CaMK2α, Ca2+/calmodulin-dependent protein kinase II alpha; CREB, cAMP response element-binding protein; ROS, reactive oxygen species; PINK1, PTEN-induced putative kinase 1; Prot, protein; RPE, retinal pigment epithelium; ER, endoplasmic reticulum; IRE1, inositol-requiring protein-1; XBP1, X-box binding protein 1; IKK, IκB Kinase; JNK, C-Jun N-Terminal Kinases

Critical pathological features of DR, such as increased vascular permeability, retinal hypoxia, and neuronal degeneration, are driven by the dysregulation of cellular processes like autophagy, mitochondrial dysfunction, ER stress and cellular senescence contributing to retinal damage. PTMs play a crucial role in regulating these processes. This section explores the mechanistic role of PTMs in these cellular dysregulations in DR (Fig. 6).

### Autophagy pathway

Autophagic/lysosomal degradation is a crucial cellular response to stress, in which autophagosomes carrying cytoplasmic components are delivered to lysosomes for degradation [190]. This process is crucial for maintaining protein homeostasis and overall cellular health. However, in DR, disruption of the internal homeostasis leads to the dysregulation of various PTMs, which exacerbates autophagy dysfunction.

Autophagic flux blockade due to high glucose in retinal neurons marks an early step in DR neurodegeneration [191]. Optineurin (OPTN) is a recently discovered autophagy receptor [192], PTM of which is closely related to the autophagy process. OPTN contribute to both autophagosome formation [193, 194] and autophagosome-lysosome fusion stage in the autophagic flux [195]. In the retina of rats with diabetes, succinylation of the K108 site of OPTN was found to mediate the blockade of the autophagy pathway under high glucose conditions [196]. When autophagic flux is compromised, the function of shuttling the ubiquitinated substrates to autophagy degradation through ubiquitin binding–associated (UBA) domain and LC3 interacting region of p62/SQSTM1 would be destroyed, leading to p62/SQSTM1 cargo accumulate, finally resulting in neurodegenerative disorders in DR (including Müller cell apoptosis and massive VEGF release) [191, 197].

Diabetes is characterized by PTMs (e.g., glycosylation and oxidation) of plasma lipoproteins, such as low-density lipoproteins (LDL). The retina, as a highly specialized tissue, has the BRB to prevent the extravasation of plasma components such as lipoproteins. The barrier is weakened in DR, causing glycated LDL to leakage, which result in RPE damage, finally increasing inflammation, oxidative stress, ER stress and autophagy [198]. As a modulator of the inflammatory response, X-box binding protein 1 (XBP1) could regulate angiogenesis, thus increasing the proliferation of endothelial cells [199], and induce autophagy via acetylation of ATG9 [200]. Above autophagic responses may be triggered by ER stress, facilitating the degradation of damaged proteins. However, prolonged ER stress can cause autophagy to shift from a protective process to one that promotes cell death [198]. Therefore, targeting the XBP1/autophagy axis in endothelial cells cannot be simply labeled as a protective strategy. While properly maintaining XBP1-induced autophagy helps endothelial cells survive early metabolic stress, over-activating XBP1 risks triggering harmful endothelial proliferation and abnormal angiogenesis. Consequently, any therapeutic intervention must carefully balance these opposing effects within the retinal vasculature rather than blindly enhancing XBP1 activity.

DR also commonly manifests as retinal ischemic changes [201], which elevate oxidative stress and trigger cell death. Under normal oxygen level, HIF-1α subunits undergo rapid degradation via the ubiquitin–proteasome pathway following hydroxylation of proline residues by HIF prolyl-4-hydroxylases (PHDs) [202]. Under hypoxic conditions, reduced PHDs activity stabilized HIF-1α, leading to its translocation to the nucleus where it induces BNIP3/NIX transcription and triggers mitophagy [203]. As a selective subtype of autophagy, mitophagy involves the targeted degradation of mitochondria and transportation to lysosomes [204]. However, the regulation of this pathway is highly dynamic throughout DR progression. In a STZ-induced DR model, excessive mitophagy driven by enhanced phosphorylation of cAMP response element-binding protein in retina tissue directly contributes to retinal neuronal degeneration [205]. Conversely, at advanced stages of neurovascular injury in the diabetic retina, mitochondrial turnover completely disrupts because both mitochondrial biogenesis and PTEN-induced putative kinase 1 (PINK1)-mediated mitophagy are impaired, where inactive PINK1 fails to phosphorylate ubiquitin at Ser65 on outer mitochondrial membrane proteins [206], resulting in toxic buildup of damaged mitochondria [207]. Hence, the pathological role of mitophagy in DR is tightly dictated by distinct PTM events across different disease stages. This temporal shift demonstrates that the therapeutic targeting of mitophagy must be stage-specific and precisely tailored to the underlying PTM state.

### Mitochondrial dysfunction

Mitochondrial dysfunction is central to the pathogenesis of DR [208, 209]. Under any pathological condition, mitochondrial dysfunction elevates nitric oxide stress, triggers PTMs of mitochondrial proteins, and can lead to oxidative damage to mitochondrial DNA [210]. The hyperglycemic milieu promotes palmitoylation of Ras-related C3 botulinum toxin substrate 1 (Rac1) [211] and impairs cytosolic antioxidant defense, leading to ROS accumulation, which damage the mitochondrial membrane, proteins, and DNA, ultimately stimulating apoptosis and exacerbating DR. In addition, hyperglycemia induces excessive production of mitochondrial superoxide, thereby activating the hexosamine pathway, which increases O-GlcNAcylation of the transcription factor SP1 and reduces its serine/threonine phosphorylation, thereby activating gene expression that leads to the onset of diabetic complications [212]. Another study has found that in diabetic rats, ubiquitination of mitochondrial transcription factor A (TFAM) in the retina hinders its transport to mitochondria, impairing mitochondrial DNA transcription and function, while inhibiting ubiquitination can restore mitochondrial homeostasis [213]. Although phosphorylation of TFAM can also reduce its protein levels in mitochondria and transcriptional activity [214], its role in DR requires further investigation.

In addition to direct glucose toxicity, hyperglycemia-induced lipid peroxidation generates reactive aldehydes that further amplify mitochondrial damage in specific retinal layers. Studies using ARPE cells have shown that hyperglycemia leads to elevated acrolein levels [215], which increases oxidative damage through protein carbonylation [216]. In DR patients, elevated levels of protein carbonyl were also found [217, 218]. While acrolein-induced mitochondrial dysfunction has been confirmed in rat brains [219] and RPE cells [215, 220], its downstream molecular footprint, acrolein-induced carbonylation of mitochondrial proteins, has been demonstrated as a mechanism in extra-retinal neuronal-like PC12 cells [221]. Whether acrolein explicitly causes the carbonylation of mitochondrial proteins within retinal neurons, endothelial cells, or Müller cells to drive retinal injury in DR remains to be further confirmed.

Crucially, the role of Müller cells in DR highlights the intersection of organelle cross-talk and PTM signaling. High-glucose stimulation of Müller cells enhances the formation of mitochondria-associated ER membranes (MAM), the physical contact point between the ER and mitochondria, which mediates Ca^2+^ flow from the ER into the mitochondria [222], resulting in mitochondrial Ca^2+^ overload, mitochondrial permeability transition pore (mPTP) opening, and functional collapse [223]. Drp1, which as regulates mitochondrial fission and stabilizes ER-mitochondrial contacts [224], undergoes activating phosphorylation at Ser616 under hyperglycemic conditions [225] under high glucose conditions [226]. Following mitochondrial damage, mtDNA is released into the cytoplasm, where it binds to cGAS to initiate cGAMP production. This induces the phosphorylation of STING, which in turn triggers the recruitment and phosphorylation of TANK-binding kinase 1, which further phosphorylated interferon regulatory factor 3 (IRF3) and NF-κB. Phosphorylated IRF3 and NF-κB are translocated into the nucleus and mediates the expressions of inflammatory factors (IFN-β and IL-6). Ultimately, this promotes inflammatory responses in DR rats, exacerbating retinal injury [226]. Therefore, blocking the interaction between ER and mitochondria, along with inhibiting Ca^2+^ transfer from ER to mitochondria, plays a key role in alleviating DR by to prevent mitochondrial dysfunction.

These findings emphasize that mitochondrial dysfunction in DR is not a homogeneous pathogenic event but an aggregate of cell-type-specific PTM disruptions. Metabolic toxicity selectively induces protein carbonylation in RPE cells to cause outer barrier damage, whereas high-glucose cross-talk fires a precise pro-inflammatory phosphorylation cascade (from Drp1 to the cGAS-STING axis) within Müller cells. Thus, effective DR interventions must be tailored to specific PTM nodes within their exact cellular environments.

### Endoplasmic reticulum stress

The ER orchestrates protein translation, production and translocation [227]. An overwhelming accumulation of misfolded proteins and calcium forces the ER into a pathological state termed ER stress, a key driver of DR that promotes BRB breakdown, pathological neovascularization, and neuronal damage. In this subsection, we dissect how specific PTM events fine-tune ER stress signaling to dictate cellular fates in the diabetic retina.

ER stress triggers the unfolded protein response (UPR) signaling pathway, which counteracts the disruptive activity via upregulating the expression of molecular chaperones and folding enzymes to alleviate the load of unfolded or misfolded proteins and restore protein homeostasis [228]. PTMs serve as key factors in protein aggregation, affecting three primary UPR sensors, including inositol requiring enzyme 1 α (IRE1α), protein kinase R-like endoplasmic reticulum kinase (PERK), and activated transcription factor 6 α (ATF6α). Alteration in these UPR sensors disrupt proteostasis and increase ER stress [229]. In normal conditions, PERK alleviates protein load by inhibiting translation, while IRE1α and ATF6 facilitates the removal of unfolded proteins by inducing the transcription of UPR target genes and chaperones [230]. Specifically, PERK phosphorylation leads to the phosphorylation of eukaryotic initiation factor 2 α (eIF2α), ultimately resulting in ATF4 expression. IRE1α activation recruits XBP1, apoptosis signal-regulating kinase 1/c-Jun N-terminal kinase (ASK1-JNK), and TNF receptor-associated factor 2 (TRAF2) to regulate PTM gene expression. Finally, ATF6 is released from the Golgi body and undergoes degradation mediated by Specific Protein 1 (SP1) and SP2, releasing its cytosolic domain. However, under chronic ER stress conditions (e.g., diabetes or viral infections), PERK and IRE1 branches can switch to activating pro-apoptotic pathways [231, 232].

During DR progression, ER stress responses diverge among retinal cell types, governed by distinct PTM modifications. In diabetic mice, XBP1 (a key component of the UPR) deficiency within retinal Müller glia resulted in compensatory surge of inflammatory and angiogenic factors, including TNF-α and VEGF, alongside elevated ER stress markers (such as ATF4/6, GRP-78, and eIF2α), ultimately leading to pronounced vascular leakage [233]. Concurrently, in retinal endothelial cells, XBP1 is also involved in regulation of inflammation activated by ER stress [234]. Interestingly, spliced XBP1, an activated form during ER stress, can be SUMOylated to elevate ER stress-induced apoptosis [235]. However, whether this specific SUMOylation switch occurs on spliced XBP1 within distinct retinal cells during DR progression, and its precise pathological weight in the ocular microenvironment, remain to be further elucidated.

In addition to glial regulation, aberrant O-GlcNAcylation within retinal endothelial cells significantly compromises barrier stability during ER stress. In retinal endothelial cells, exposure to ER stress inducers triggers the GRP78 translocation to the plasma membrane, which increases O-GlcNAcylation of proteins, such as focal adhesion kinase (regulating vascular permeability), cathepsin D (affecting endothelial permeability), and especially VE-cadherin, thus resulting in impaired complex formation [236]. Simultaneously, metabolic signaling drives a separate pathogenic phosphorylation cascade within these same endothelial elements. Hyperglycemia elevates the activity of 12/15 lipoxygenase, generating bioactive lipid products (12- and 15- hydroxyeicosatetraenoic acid) that induce intracellular calcium influx. This calcium disturbance fires ER stress, manifested as phosphorylated PERK and ATF6, which subsequently activate nicotinamide adenine dinucleotide phosphate (NADPH) oxidase and stimulates the downstream phosphorylation of VEGFR2, culminating in profound retinal endothelial dysfunction [237]. Together, these pathways demonstrate that ER stress concurrently exploits aberrant O-GlcNAcylation to tear down structural junctions and hyper-phosphorylation to amplify downstream endothelial signaling.

In contrast to these vascular events, ER stress-mediated neuronal damage in the diabetic retina is dictated by the failure of a specific ubiquitination clearance system. The homeostatic removal of misfolded proteins relies on ER-associated degradation (ERAD), powered by the coordination between the E3 ligase HRD1 and the ATP-dependent chaperone regulating the system VCP/p97). In DR, reduced expression of deubiquitination enzymes (LCB3) and VCP/p97 indicates a compromised ubiquitin–proteasome system and diminished ERAD efficiency, leading to retinal endothelial cell and pericytes damage [238]. Understanding the interplay between PTMs and ER stress is essential for uncovering new therapeutic targets aimed at restoring protein homeostasis and preventing disease progression.

### Ageing

Ageing can be defined as a biological process that causes damage and deterioration in function of tissues and organs in the body, thereby increasing susceptibility to various diseases and the risk of death. As with other organs, ageing alters the structure of the retina, including decreased overall retinal thickness, a 20% reduction in macular blood flow, decreased reactivity of microglia to injury, and their migration to the outer retina, promoting inflammation [239, 240]. At the same time, ageing induces metabolic changes in the retina, leading to the accumulation of AGEs in the RPE and Bruch’s membrane [241]. Methylated metabolites such as 1-methylnicotinamide (1-methylNAA), 3-methylglutaric acid, and 5ʹ-methylthioadenosine accumulate in the ageing retina [242]. Ageing is a major risk factors for retinal degenerative diseases [243, 244]. One Study has found that the prevalence of DR increases with age, particularly between 55 and 74 years [244].

During the ageing process, ROS initially stimulate damage to mitochondrial, leading to increased ROS production in mitochondria, which in turn causes further mitochondrial damage, forming a vicious cycle that results in mitochondrial dysfunction [245]. Therefore, mitochondrial dysfunction is one of the major features of ageing, leading to increased susceptibility to DR. In addition, genomic instability is another characteristic of ageing. Montesanto et al. found significant associations of variants in the *CAT*, *FTO*, and *UCP1* genes with DR [246]. Similarly, a report indicates that enhanced oxidative DNA damage in diabetes is an important factor in the pathogenesis of diabetic complications [247]. Hyperglycemia can also induce DNA damage by AGEs [248]. Therefore, genomic instability is another feature linking ageing to DR risk factors.

Telomere attrition is another important factor contributing to ageing. Telomeres are repetitive non-coding DNA sequences that safeguard the ends of chromosomes from damage. Telomere shortening also accelerates the process of ageing, as it has been observed that telomere length shortens with age. Previous researches suggest that telomere attrition may serve as a marker for the presence and extent of diabetes complications [249, 250], providing additional prognostic insight into mortality risk in patients with type 2 diabetes [251]. Interestingly, the association between telomere shortening and ageing is more closely related in DR patients [252].

Moreover, cell senescence, characterized by permanent cessation of cellular growth [253], can also be observed during ageing. During ageing, DNA damage, mitochondrial dysfunction, and telomere attrition can stimulate cell senescence [254]. Many studies have revealed the role of endothelial cell senescence in the development of DR, particularly in the formation and regression of pathological neovascularization. For example, Crespo-Garcia et al. identified the presence of senescent vascular cells in the retinas from postmortem eyes of patients with proliferative diabetic retinopathy (PDR) [255]. In vivo, premature senescence of retinal endothelial cells has also been observed in STZ-induced hyperglycemic mice, as indicated by increased mRNA levels of senescence markers [255]. Therefore, we can see that mitochondrial dysfunction, genomic instability, telomere attrition, and cellular senescence are major hallmarks of ageing and are also associated with neurodegeneration, indicating that ageing is indeed an important risk factor for DR.

In addition, various PTMs act as critical chronological rheostats regulating cellular senescence during DR progression. Inside the outer retinal barrier, high-glucose stress the mitochondrial enrichment of TRF1-interacting protein 2 (TIN2) within the RPE mitochondria. This mitochondrial accumulation of TIN2 sequentially induces phosphorylation of Forkhead box O1 (FOXO1) and its translocation into the mitochondria, thereby suppressing mitochondrial respiration and impairing cellular antioxidant defense capacity, finally exacerbating mitochondrial damage and accelerating RPE senescence [256, 257]. Concurrently, within the inner BRB, cellular aging is governed by a distinct PTM-mediated degradation pathway. Chronic metabolic stress activates the cyclic GMP-AMP synthase (cGAS)/stimulator of interferon genes (STING) axis in retinal endothelial cell, which elevated p53 activity, promoting the ubiquitination and degradation of FOXO3a to aggravate endothelial senescence [258, 259]. This senescence landscape further extends to the neurosensory layers, where the loss of homeostatic PTM shielding drives neurodegeneration. Senescence marker protein 30 (SMP30), an ageing-related protein, is significantly downregulated in RGC high glucose stimulation. Crucially, this diminished SMP30 expression leads to reduced phosphorylation of Akt and glycogen synthase kinase-3β (GSK-3β), ultimately triggering RGC apoptosis, inflammatory responses, and oxidative stress [260]. With these results, it becomes clear that cellular senescence in DR is not a uniform degenerative process, but a localized pathology tightly partitioned by cell-specific PTM signatures. Deciphering this programmatic PTM shift, from targeted protein degradation in the vasculature to alternative kinase activation in neurons, is essential for developing targeted anti-aging interventions in the diabetic retina.

## Targeted at PTMs as a therapeutic target for DR

Alteration in PTMs can lead to the aggregation of misfolded proteins, thereby increasing neurotoxicity by disrupting cellular signaling cascades. These impaired signaling pathways hinder biological processes like autophagy, mitophagy, inflammatory responses, cell cycle regulation, and mitochondrial function, resulting in retinal microenvironment disturbances. Recent studies indicate that drug and natural biomolecules targeting various PTMs are of significant importance in the treatment of DR (Table 2).

**Table 2:** The application of diverse drug and biomolecules in DR targeting various post-translational modifications

Drug	PTMs	Biological effects	Refs
Dapagliflozin	Phosphorylation	alleviated HRMECs apoptosis induced by high glucose through ERK1/2/cPLA2/AA/ROS pathway	[261]
Fenofibrate	Phosphorylation	Suppressed phosphorylated form of JNK expression and Ask-1 phosphorylation, which in turn inhibits the activation of the subsequent apoptotic signaling pathways	[262–264]
AdipoRon	Phosphorylation	activated 5ʹ AMPK/acetyl-CoA carboxylase phosphorylation downstream, thereby alleviating oxidative stress and eventual apoptosis of cells and tissues	[265]
Metformin	Phosphorylation	Activated AMPK to reverse mitochondrial dysfunction and inhibit RPE cells death	[166]
Axitinib	Phosphorylation	reduce the activation of both VEGFR1 and VEGFR2, through their reduced phosphorylation	[266]
Aflibercept	Phosphorylation	Eliminate the effect of VEGFR2 phosphorylation, which was caused by alectin-1	[267]
Chrysin	Phosphorylation	down-regulate the phosphorylation of AKT, ERK and MMP-2 and reduce the effects of VEGF and VEGFR in a high glucose environment	[268]
Capilliposide B	Phosphorylation	inhibits VEGF-stimulated activation of the VEGFR2 signaling pathway and abrogates downstream phosphorylation	[269]
Retinol binding protein 3	Phosphorylation	binding and inhibiting VEGF receptor tyrosine phosphorylation	[270]
Donepezil	Phosphorylation	improved phosphorylation of PI3K and mTOR, thus exerting a neuroprotective effect.	[271]
Mangiferin	Phosphorylation	suppressed RRCEC angiogenesis via modulating the PI3K/AKT/mTOR signaling pathway	[272]
N6-furfuryladenine (kinetin)	Phosphorylation	amplify mitophagy in a PINK1-dependent fashion over its precursor kinetin, enhancing mitochondrial turnover and offering neuroprotection	[273–275]
Liraglutide	Phosphorylation	reverse hyperphosphorylated tau-triggered synapse loss in diabetic retinae	[276]
Tofacitinib	Phosphorylation	reduced phosphorylated form of JAK1 expression, thus reducing retinal albumin leakage	[186]
Agmatine	Phosphorylation	protects Müller cell form glucose-induced damage and offer protection against BRB breakdown	[277, 278]
Genipin	Phosphorylation Glycosylation	protects cells and tissues from high glucose levels by regulating phosphorylation and glycosylation	[279]
Glucosamine	Glycosylation	anti-inflammatory effects	[280]
TUDCA	O-GlcNAcylation	protects cells from DR damage	[281]
Trichostatin A	Ubiquitination	reduced Nox4 expression and angiogenesis via ubiquitination of p300- histone acetyltransferase	[282]
Betulinic acid	Proteasome Activator	Rescue of these pathological processes	[283]
Resveratrol	Acetylation	plays an anti-inflammatory role in DR, as well as inhibit retinal angiogenesis and oxidative stress	[284]
Ginsenoside Rd	Deacetylation	protect retinal endothelial from injury	[285]
Phloretin	Deacetylation	effectively attenuate PA-induced oxidative stress in endothelial cells	[286]
Hawthorn polyphenols	Acetylation	significantly reduced acetylated NF-κB p65 expression	[169]
Minocycline	Methylation	normalize the altered histone methylation levels, playing beneficial effect on the retinas of diabetic rodents	[94]
Tempol	Nitration	preserve the integrity of BRB	[287]
Aminoguanidine	Nitration	Inhibited nitration of a retinal protein	[288]
Calcium dobesilate	Nitration	prevent the BRB breakdown induced by diabetes	[289]
Sitagliptin	Nitration	prevent diabetes-induced retinal protein nitration	[290]
Epicatechin	Nitration	Block tyrosine nitration of PI3K	[291]

PTMs, post-translational modifications; HRMECs, human retinal microvascular endothelial cells; ERK1/2, extracellular signal-regulated kinase 1/2; cPLA2, cytosolic Phospholipase A2; AA, arachidonic acid; ROS, reactive oxygen species; JNK, c-Jun N-terminal kinase; ASK-1, apoptosis signal-regulated kinase-1; AMPK, adenosine monophosphate-activated protein kinase; RPE, retinal pigment epithelial; VEGFR1/2, vascular endothelial growth factor receptor 1/2; MMP-2, matrix metalloproteinase 2; PI3K, phosphoinositide 3-kinase; mTOR, mammalian target of rapamycin; RRCEC, rat retinal endothelial cells; PINK1, PTEN-induced putative kinase 1; JAK1, Janus kinase 1; BRB, blood-retinal barrier; TUDCA, tauroursodeoxycholic acid; DR, diabetic retinopathy; Nox4, Nicotinamide adenine dinucleotide phosphate oxidase 4; PA, palmitic acid

### Targeting phosphorylation

Dapagliflozin decreases the production of arachidonic acid (AA) and inhibits ERK1/2 phosphorylation, which in turn attenuates cytosolic phospholipase A2 (cPLA2) phosphorylation, ultimately alleviating HRMECs apoptosis [261]. Two large randomized clinical studies have shown that the peroxisome proliferator-activated receptor α (PPAR-α) agonist fenofibrate can prevent DR progression [262, 263]. Specific studies revealed that fenofibrate inhibits the phosphorylation of JNK and apoptosis signal-regulated kinase-1(ASK-1), protect against oxidative stress-induced apoptosis in retinal/choroidal endothelial cells [264]. After oral administration of AdipoRon (an adiponectin analog) to STZ-induced diabetic mice, it binds to adiponectin receptor 1 on Müller cells. Then it activated 5ʹ AMPK/acetyl-CoA carboxylase phosphorylation downstream, thereby alleviating oxidative stress and eventual apoptosis of Müller cells in high glucose environment [265]. As an AMPK agonist, metformin could inhibit RPE death and reverse mitochondrial dysfunction via promoting AMPK phosphorylation [166]. From a clinical management perspective, these metabolic-regulatory agents offer significant translational potential by shifting the therapeutic focus from late-stage microvascular destruction to upstream neuroglial preservation. Specifically, modulating AMPK or JNK phosphorylation kinetics in Müller and RPE cells represents a promising strategy for proactive neuroprotection, potentially allowing clinicians to mitigate retinal cellular stress before irreversible structural damage manifests on conventional fundus imaging.

When angiogenic factors binds to VEGFR, it would cause ERK protein phosphorylation, subsequently exacerbating angiogenesis and inflammation in DR [292, 293]. As a VEGFR inhibitor, axitinib reduces the activity of VEGFR1 and VEGFR2 by decreasing their phosphorylation, and also reduces the activity of the ERK pathway, thereby protecting retinal endothelial cells and slowing the progression of DR [266]. Application of Alectin-1 to HRMECs enhanced VEGFR2 phosphorylation, which was reversed by aflibercept [267]. In addition, chrysin [268], Capilliposide B [269], and Retinol binding protein 3 [270] can all inhibit the phosphorylation of VEGFR. In clinical practice, while current anti-VEGF therapies successfully suppress advanced macular edema and neovascularization, their repetitive intravitreal administration carries risks of severe complications. Mechanistically targeting VEGFR phosphorylation pathways via these novel small molecules or natural compounds provides an alternative approach to stabilize the BRB and inhibit pathological angiogenesis, which may eventually reduce injection frequencies and optimize long-term patient compliance.

The mechanism of retinal apoptosis is controlled by the PI3K/Akt/mTOR pathway, which has been reported to afford protection of RGC [294] and RPE cells [295]. The use of Donepezil (an acetylcholinesterase inhibitor) can upregulate the phosphorylation of PI3K/mTOR, thereby exerting a neuroprotective effect [271]. Differently, mangiferin downregulates the phosphorylation of PI3K/mTOR, thereby inhibiting high-glucose-induced angiogenesis in rat RCEC, which can serve as an effective treatment strategy for DR [272]. N6-furfuryladenine (kinetin) acts as an effective PINK1 kinase activator, leading to the phosphorylation of ubiquitin (at Ser65) conjugated to outer mitochondrial membrane proteins [273, 274], thereby safely rescuing mitochondrial turnover and exerting a neuroprotective effect in the diabetic mouse retina [275]. Moreover, liraglutide could reverse hyperphosphorylated tau-triggered synaptic degeneration of RGCs in diabetic retina [276]. Importantly, the dual roles of the PI3K/Akt/mTOR and PINK1 pathways highlight a critical clinical gap: a single phosphorylation cascade can act as a survival signal for RGCs but a pathogenic driver for endothelial proliferation. Therefore, future translational applications must balance these complexities, utilizing cell-type-specific modulators to achieve targeted neuroprotection without inadvertently accelerating pathological vascular leakage.

Tofacitinib treatment in db/db mice for two weeks could reduce the expression of phosphorylated JAK1, ameliorating BRB dysfunction and retinal albumin leakage [186]. Studies have shown that indicating that agmatine is able to protect against BRB breakdown primarily by reducing VEGF protein levels through inhibition of NMDAR/Ca^2+^ signaling and its downstream MAPK pathway [277]. Similarly, Chronic agmatine treatment similarly exerts a neuroglial protective effect by reducing phosphorylation of ERK, JNK, and p38 in high glucose-induced Müller cell damage [278]. These findings indicate that preventing the hyperphosphorylation of downstream MAPK and JAK/STAT cascades holds critical prognostic and therapeutic value.

### Targeting other PTMs

Genipin increased HIF-1α phosphorylation and decreased intracellular glycosylation, thus ameliorating retinal microangiopathy [279]. Glucosamine inhibits the expression and function of ICAM-1 by regulating the O-linked glycosylation of NF-κB signaling factors and reducing the N-linked glycosylation of TNF-α-induced ICAM-1 in ARPE-19 cells, thereby exerting anti-inflammatory effects [280]. Tauroursodeoxycholic acid (TUDCA) protects against DR damage by inhibiting VE-cadherin O-GlcNAcylation, blocking ER-induced apoptosis, and reducing VEGF production and vascular leakage [281]. These evidences suggest that targeting specific glycosylation and O-GlcNAcylation events offers a highly effective therapeutic strategy to prevent BRB and chronic macular edema. Since glycosylation dynamically regulates a vast array of essential proteins throughout the retina, broad and unguided interventions into these pathways risk disrupting homeostatic cellular metabolism. Consequently, future research should transition from pan-glycosylation modulation toward identifying site-specific modified links, ensuring that therapeutic agents can precisely rescue vascular endothelial junctions without inadvertently compromising overall retinal cell survival.

Trichostatin A can reduce NADPH oxidase 4 (Nox4) expression and angiogenesis by ubiquitinating p300-histone acetyltransferase, which can be used to prevent abnormal angiogenesis in DR [282]. Betulinic acid, a proteasome activator, can rescue of the pathological processes caused by severe proteasome dysfunction resulting from methylglyoxal-H1 accumulation, including glucose homeostasis disorders, which further exacerbate angiogenic activation of the hyaloid and retinal vasculature [283]. Consequently, manipulating the ubiquitin-proteasome system to accelerate the clearance of toxic protein aggregates offers a shift in DR therapeutics.

Resveratrol activates SIRT1/HMGB1 (high mobility group box 1), thereby causing SIRT1 to regulate HMGB1 acetylation, which inhibits inflammation, angiogenesis, and oxidative stress in DR [284]. Ginsenoside Rd strengthened the interaction between AMPK and SIRT1 by increasing NAD+/NADH levels and Tumor suppressor liver kinase B1 (LKB1) deacetylation in endothelial cells, therefore, it beneficially modulated oxidative stress and apoptosis, and ameliorated diabetes-driven vascular damage [285]. Phloretin could restore the activity of manganese superoxide dismutase (MnSOD) through deacetylation in vitro and in vivo, and then obviously reduced the accumulation of mitochondrial ROS [286]. Hawthorn polyphenols significantly reduced acetylated NF-κB p65 expression, thus alleviating hyperglycemic ARPE-19 inflammation [169]. Minocycline treatment can restore histone methylation levels in the retinas of diabetic rats to normal levels [94]. These findings demonstrate that using natural polyphenols and established multi-target compounds to modulate acetylation and methylation states offers a distinct advantage in DR management. However, their inherently poor ocular bioavailability and rapid systemic metabolism limit the application. Therefore, optimizing these PTM-modulating agents requires future clinical research to focus on structural chemical modifications or specialized ocular drug-delivery vehicles to overcome this pharmacokinetic bottleneck.

Chronic administration of tempol reduces the levels of oxidized LDL and glycated LDL in the retina, 3-nitrotyrosine formation, PGIS nitration, and retinal cell apoptosis, thus protecting the integrity of BRB [287]. Aminoguanidine can also inhibit the nitration of retinal proteins in diabetes [288]. Similarly, treatment with Calcium dobesilate can prevent the increase in tyrosine residue nitration in diabetic retina, thereby exerting a protective effect on BRB permeability [289]. Furthermore, sitagliptin can prevent diabetes-induced nitration of retinal proteins [290]. Epicatechin could block tyrosine nitration of the p85 subunit of PI3K to protect early retinal vascular cell death and late neovascularization [291]. Nevertheless, a major hurdle for these anti-nitration strategies is the lack of tissue specificity, as peroxynitrite formation and protein nitration are fundamental to systemic physiological signaling. Future therapeutic approaches must pivot away from non-specific systemic administration and prioritize localized, retina-targeted delivery, ensuring these protective anti-nitration effects are restricted to damaged retinal cells without disrupting systemic homeostasis.

## Summary and future prospects

In summary, the main characteristic of DR lies in abnormal protein expression, functional disorders, and alterations in PTMs triggered by hyperglycemia and metabolic disturbances. Decades of research have indicated that multiple types of PTMs, including phosphorylation, glycosylation, methylation, acetylation, ubiquitination, and SUMOylation, may be involved in the pathogenesis of DR. However, aberrant PTM dynamics disrupt the intrinsic functional orientation and structural stability of pivotal signaling hubs, such as the PI3K/Akt, MAPK, AMPK, NF-κB, and JAK/STAT pathways, thereby dysregulating protective autophagy and mitophagy, inducing cell cycle disorders, compounding mitochondrial dysfunction, and aggravating ER stress. These molecular deviations ultimately damage the tight junctions of BRB, lead to retinal degenerative alterations and accelerate the progression of DR. Thus, there is a growing need for therapeutic molecules targeting different types of PTMs to inhibit misfolded protein aggregation or promote the clearance of toxic aggregates. Consequently, targeting these diverse PTM nodes to inhibit misfolded protein aggregation or to facilitate the clearance of toxic macromolecular aggregates represents a critical and expanding therapeutic imperative.

However, moving these preclinical discoveries into clinical practice poses major challenges, as the exact clinical orientations and specific mechanisms of PTMs in DR remain partially obscured. The primary challenge is the lack of tissue specificity, because most core PTM-catalyzing enzymes are ubiquitously expressed and essential for general systemic homeostasis, broad systemic inhibition carries high risks of off-target toxicities. Therefore, future research in the DR management field should urgently prioritize several promising avenues. On one hand, future investigations need to utilize localized, retina-targeted drug delivery platforms, such as functionalized nanoparticles or sustained-release ocular implants, to ensure that these PTM-modulating agents operate exclusively within the ocular microenvironment, thereby maximizing therapeutic concentration at the posterior segment. On the other hand, investigating the precise, cell-type-specific roles of PTMs across distinct retinal layers to minimize unwanted side effects on neurons or glia is necessary. By addressing these practical delivery and target-specificity bottlenecks, future research on the retinal PTM landscape will provide deeper insights for both the early detection and individual management of DR.

## Data Availability

No datasets were generated or analysed during the current study.
